# A Robotic Testing Platform for Pipelined Discovery of Resilient Dielectric Elastomer Actuators

**DOI:** 10.1002/advs.77008

**Published:** 2026-08-11

**Authors:** Ang (Leo) Li, Alexander Yin, Alexander White, Sahib Sandhu, Anatol Gogoj, Denise Maria de Andrade, Matthew Francoeur, Victor Jimenez‐Santiago, Van Remenar, Codrin Tugui, Mihai Duduta

**Affiliations:** ^1^ Department of Mechanical and Industrial Engineering University of Toronto Toronto Canada; ^2^ Institute of Materials Science University of Connecticut Storrs USA; ^3^ School of Mechanical, Aerospace, and Manufacturing Engineering University of Connecticut Storrs USA; ^4^ Material Science and Engineering University of Connecticut Storrs USA; ^5^ Inorganic Polymers Department Petru Poni Institute of Macromolecular Chemistry Iasi Romania

**Keywords:** dielectric elastomer actuator, self‐driving laboratory, soft robotics

## Abstract

Short lifetime under high electrical fields hinders the widespread robotic application of linear dielectric elastomer actuators (DEAs). Systematic scanning is difficult due to time‐consuming per‐sample testing and the high‐dimensional parameter space affecting performance. To address this, we propose a parameter screening pipeline enabled by a novel testing robot capable of scanning DEA lifetime. The robot integrates electro‐mechanical property measurement, programmable voltage input, and multi‐channel testing capacity. Using it, we scanned the lifetime of Elastosil‐based linear actuators across parameters including input voltage magnitude, frequency, electrode material concentration, and electrical connection filler. The selected parameter combinations improved operational lifetime under boundary operating conditions by up to 100% and were subsequently scaled up to achieve higher force and displacement output. The final product demonstrated resilience on a tethered modular, scalable quadruped walking robot prototype with payload carrying capacity (>100% of its untethered body weight, and >700% of combined actuator weight). This work introduces a robotic DEA testing platform and demonstrates a systematic DEA lifetime‐scanning pipeline, providing a foundation for future device‐level self‐driving laboratory development.

## Graphical abstract

This work introduces a robotic testing platform enabling high‐throughput, multi‐parameter lifetime mapping of linear dielectric elastomer actuators (DEAs). By integrating programmable high‐voltage control and automated electromechanical measurements, the system identifies material and operating conditions that double actuator lifetime. Optimized actuators are scaled and deployed in a modular quadruped robot demonstrating unprecedented payload capacity for DEA‐driven locomotion.

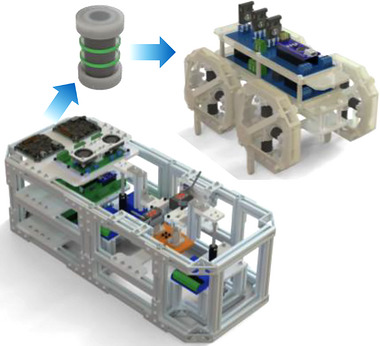

## 1 Introduction

Reduced lifetime under high electrical fields limits the practical use of linear dielectric elastomer actuators (DEAs) in aerial [1, 2], aquatic [3, 4], walking [5, 6], and inspection [7, 8] robots. While most actuator designs achieve long lifetimes at low electrical fields, their durability drops sharply under higher fields. Yet, maximum power, strain, and force outputs are realized only near high‐field operation (80% of the breakdown field) due to the near‐exponential dependence of mechanical output on electric field strength [9]. Although the community commonly reports DEA longevity in terms of “cycle life”, we find that “lifetime” is a more meaningful metric, since high‐frequency actuation can yield large cycle numbers that do not accurately reflect material degradation [10]. Despite its critical importance, DEA lifetime research is challenging: obtaining such data is time‐intensive, requiring prolonged per‐sample tests that occupy valuable instruments, such as high‐voltage power supplies, oscilloscopes, function generators, and force or displacement sensors. Furthermore, lifetime spans a multidimensional parameter space, including voltage, frequency, material composition, and processing conditions. The high resource demand and complexity make systematic lifetime mapping impractical using conventional manual testing approaches.

Developing stand‐alone, high‐throughput, and modular testing instruments is fundamental to advancing DEA lifetime research. Previous DEA testing platforms have established important capabilities for multi‐channel lifetime characterization, programmable actuation, and environmental‐condition testing [10, 11, 12, 13, 14]. As summarized in Figure S1, these developments have enabled systematic studies of degradation behavior and the evolution of electrical properties throughout operation [15, 16]. Building on this foundation, the next step is to treat DEA lifetime as a multidimensional design space by linking high‐throughput electromechanical lifetime testing, systematic scanning of material and operating parameters, and downstream integration of improved actuators into functional robotic systems. Advances in self‐driving labs (SDLs) have demonstrated the power of robotic experimental platforms and algorithms to automate data‐intensive, high‐dimensional, and time‐consuming research [17], ranging from optimizing digital composites [18] to high‐throughput semiconductor material discovery [19] and characterization [20]. Partial SDL implementations have also advanced battery cycle‐life prediction using machine learning algorithms on collected dataset [21], and honeybee colony monitoring through autonomous robotic data collection [22]. Building on these developments, this work adopts a self‐driving‐lab‐inspired approach to introduce robotic testing infrastructure into DEA lifetime research. This work is not presented as a fully autonomous or closed‐loop SDL system, but rather as a step toward such a long‐term research direction. The contributions of this work are: (1) the development of a high‐throughput DEA testing robot, (2) a device‐level research pipeline for systematic lifetime studies, and (3) a research vision toward future closed‐loop and autonomous DEA design platforms.

**FIGURE 1:**
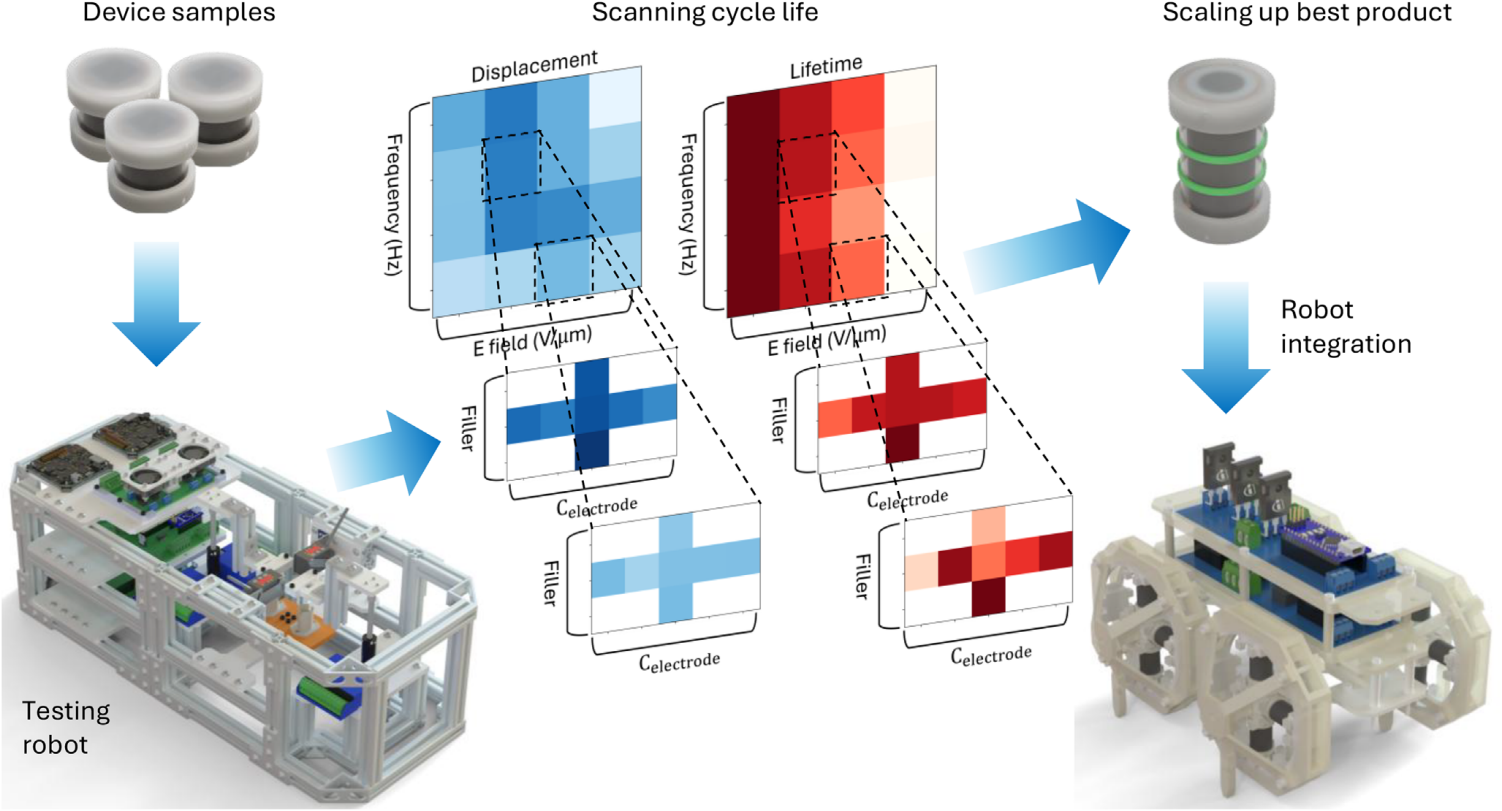
Linear DEA life‐time screening pipeline Batches of linear DEA samples are fabricated and tested using the DEA testing robot. lifetime is scanned across the parameter space defined by voltage, frequency, and material choices. The optimal parameter combination is then used to scale up the actuator in size and layer number, achieving higher displacement and force output. Finally, the optimized actuators are integrated into a quadruped walking robot.

In this work, we propose an life‐time screening pipeline for geometrically linear DEAs that spans from parameter scanning to downstream integration of optimized actuators into mobile robots. The pipeline described in Figure 1 is enabled by a novel testing robot that integrates multi‐channel programmable voltage input and electromechanical measurement capabilities, including current, voltage, impedance, displacement, and blocked force. A stepper‐motor‐based automated system switches actuators between force and displacement measurements. Batches of simplified linear DEAs (10 active layers, 1.2 cm tall) made of Elastosil P7670 [23] elastomer are fabricated and tested using this system. The lifetime scanning procedure starts from the best‐known material configuration, samples are tested under a DC square wave to scan frequency and electric field, identifying the optimal operating range that yields the longest lifetime while maintaining strong mechanical performance.

In the next stage, actuators fabricated with varying electrode concentrations and electrical connection filler materials are tested at the identified optimal voltage field and frequency to determine the best material combinations. The structure of linear actuators and their operating principle is shown in Figure S2. Electrical connections, often overlooked in DEA research, play a critical role in device durability. We found that the conductive filler used at the electrical connection can significantly influence lifetime performance (up to 500% among materials scanned in this work). The optimal parameters identified through this pipeline are then used to fabricate scaled‐up actuators with increased layers (20 active layers), size (2 cm tall), and added reinforcement to enhance axial strain. These optimized actuators are integrated into a tethered modular scalable quadruped robot prototype. The robot demonstrates the payload capacity of >100% of its tethered body weight and >700% of combined actuator weight. The demonstration highlights the potential of compliant, largely non‐metallic electrostatic actuators for robotic operation in environments where electromagnetic motor‐driven systems may face limitations, such as strong electromagnetic‐field environments.

This work demonstrates a systematic and automated approach to investigating one of the most critical and resource‐intensive attributes of DEAs: operational lifetime. This work explores how robotic high‐throughput testing infrastructure and systematic actuator lifetime studies could support future device‐level SDL development for soft actuators. The testing robot, built from open‐source electronics and off‐the‐shelf instruments, can be easily scaled beyond two channels and serves as a data‐collection engine for DEAs. The collected data can potentially support model training for performance prediction, rapid evaluation, and material design. Future work will integrate automated sample fabrication and data‐driven optimization to evolve the system into an autonomous DEA design platform. Our vision toward a closed‐loop, device‐level SDL, using DEAs as a case example, is outlined in the discussion section.

## 2 Results

### 2.1 Testing Robot

**FIGURE 2:**
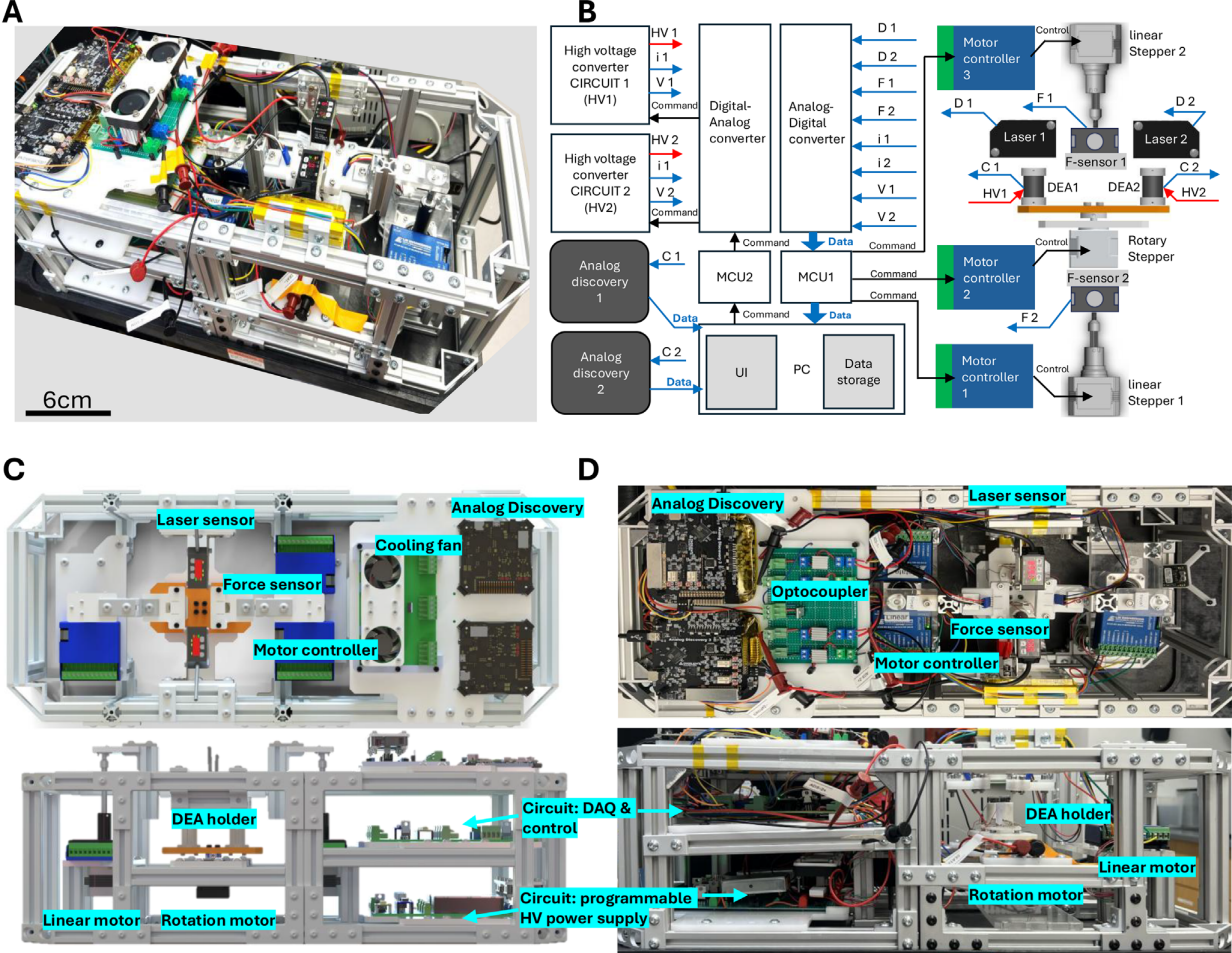
The linear DEA testing robot. **(A)** the testing robot. **(B)** the block diagram of the system architecture. **(C)** the top‐down and side views of the testing robot. **(D)** the photos of the top‐down and side views of the testing robot.

The actuator testing robot is the central enabling component of the discovery pipeline. Its design principle is to integrate a comprehensive set of electromechanical measurement capabilities into a standalone system (Figure 2A). The robot has a benchtop‐compatible physical profile, allowing easy integration into existing lab setups. Open‐source electronics and measurement instruments enhance system integration at the programming level. Key hardware components, including high‐voltage converters, laser displacement sensors (LDS), and force sensors, are mounted in a plug‐and‐play configuration, enabling quick replacement or upgrades to accommodate different power and measurement ranges.

The proposed testing robot has a two‐channel setup, where each channel has an independent power supply and measurement track, but both are controlled by a single controller set (Figure 2B). This architecture balances scalability with integrated data collection and control. For mechanical property measurement, each channel includes an LDS for displacement measurement and a force sensor for blocked‐force testing. Since displacement and blocked force cannot be measured simultaneously, a motorized switching mechanism is integrated to alternate between the two modes. A rotary stepper motor carries a plate that holds the linear DEA samples for each channel. By rotating, it positions the actuators beneath either the LDS or the force sensor. Blocked‐force measurement requires restricting actuator motion; to achieve this, the force sensor is mounted on a linear motor that automates constraining and releasing. During testing, the linear motor lowers the force sensor onto the DEA sample and uses the force reading as feedback until a predefined threshold is reached, after which the actuator is released (Movie S1).

The main electronics are organized into two stacked circuit boards: one for data collection and control, and another as a programmable high‐voltage power supply (Figure 2C,D). The top board integrates the analog–digital converter (ADC) interface for all electromechanical measurements, including displacement, force, and current–voltage feedback from the power (circuit diagram, Figure S4). It also provides motor control interfaces. A microcontroller (MCU: Arduino Nano) handles data acquisition and automatic motor control. The same MCU controls the high‐voltage opto‐couplers (HVM OR‐100), which isolate the DEAs and the LCR meters (embedded in the top‐layer circuit) from the power supply when switching measurement modes. The bottom board serves as a dual‐channel programmable power supply (circuit diagram, Figure S5). Each channel independently controls voltage magnitude, frequency, and waveform. Default high‐voltage converters are XP Power HRC series (5 W). A user interface (UI) programmed in Python enables communication between the Arduinos and the PC. The UI connects to both Arduino MCUs and manages data acquisition. When the DEAs are isolated from high voltage, the robot performs impedance measurements using two Digital Analog 3 (DA3) units. These DA3s are controlled via manufacturer‐provided APIs integrated into the UI. The overall architecture and workflow of the testing robot is shown in Figure S6.

The testing robot is designed as a standalone modular unit with USB interfaces for PC communication and an independent power connection. This standalone and modular architecture simplifies scalability by decoupling component‐level electrical constraints from system expansion, reducing the primary scaling challenges to data handling and power consumption. Importantly, scaling the number of testing channels does not require modification of the underlying testing methodology or control architecture. Larger testing setups can therefore be constructed by replicating the two‐channel units and connecting them to a centralized control interface, allowing each unit to execute independent testing protocols while aggregating data collection and storage.

Two scale‐up strategies are envisioned. In the first approach, the USB interfaces of multiple units are connected through a centralized USB hub to a single PC, while all units are powered by a shared benchtop power supply. In this configuration, scalability is primarily limited by USB data‐transfer bandwidth and the available power capacity. In the second approach, intended for high‐channel‐count and high‐throughput operation, the analog‐to‐digital converter (ADC) channels on the data‐acquisition circuit can be bypassed and connected directly to a centralized high‐channel‐count DAQ system. This architecture decouples data acquisition from protocol control and removes the USB communication bottleneck. In this case, scalability is primarily constrained by the available DAQ channels and the total power‐supply capacity.

The Analog Discovery boards used for impedance measurement function as optional low‐voltage LCR meters and are not required for continuous actuator operation. Since impedance measurements are performed discretely under isolated low‐voltage conditions, a switch board mounted on top of the testing rig (Figure 2D) enables a single Analog Discovery unit to sequentially measure impedance across multiple testing channels.

### 2.2 Dea Lifetime‐Screening Workflow

We developed and implemented a staged lifetime‐screening workflow that first scans the boundary conditions of the operating regime, including electric field and frequency, followed by material parameters to extend lifetime under these boundary conditions. Here we define the operational lifetime as the time before device displacement degrades to 80% of its initial value. Displacement was selected because actuation strain directly determines the usable mechanical output of DEA‐driven robotic systems. However, DEA degradation is inherently multi‐parameter and may additionally involve changes in electrical, mechanical, and thermal properties [10, 12, 15, 16]. The platform is therefore designed to support flexible acquisition of multiple electromechanical measurements, allowing researchers to adopt lifetime metrics most appropriate for their specific applications and research objectives. These parameters can provide important insight into degradation mechanisms, but they are indirect indicators of application‐level actuator performance compared to displacement degradation. This definition is inspired by practices in the battery industry [24] and is more practically relevant than the 50% criterion [25]. The platform can monitor electrical properties including current feedback during operation and capacitance changes before and after long‐duration testing. Measurements showed that higher actuation voltages led to greater capacitance degradation (Figure S7), indicating progressive changes in the electrical state of the actuator during aging. In addition, displacement degradation can lead changes in current feedback (Figure S8), suggesting that operational performance degradation is not always directly reflected by electrical‐current measurements alone.

The DEA samples are fabricated by rolling a multilayer DEA sheet and encapsulating it between two rigid caps for electrical connection (Figure S2). Details of the multilayer fabrication process are provided in the Materials and Methods. The material parameters explored in this work include (1) the electrical connection filler and (2) the carbon nanotube (CNT) electrode concentration. The electrical filler is the conductive material that interfaces the DEA with the electrical wire to form a reliable conductive path (Figure S2). This filler is typically a colloidal material applied by brushing or painting. Through experimental practice, we found that the choice of filler material has a significant impact on device lifetime, yet this factor has received little attention in the DEA literature.

**FIGURE 3:**
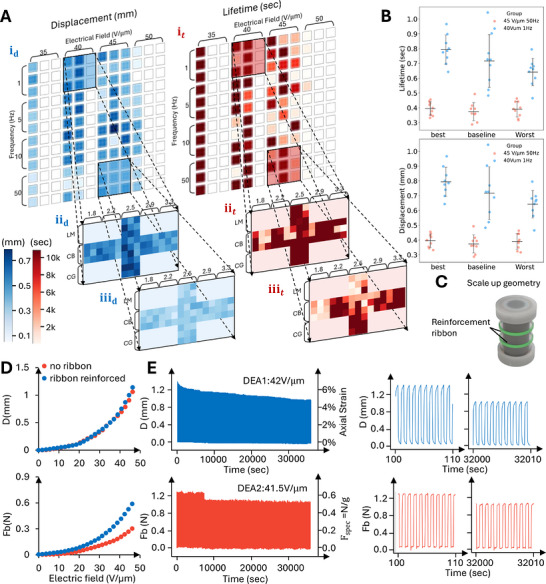
Parameter screening for optimal lifetime and mechanical output. **(A)** layered parameter screening structure. **(B)** Improvement comparison of lifetime and average displacement at 1 Hz (40,V/μm) and 50 Hz (45,V/μm) for the best, baseline (default), and worst material combinations. Points represent individual samples, while bars and error bars indicate mean and standard deviation, respectively (N=9 independent samples per condition). Pairwise two‐sided t‐tests between the best and baseline conditions yielded p=0.278 at 1 Hz (40,V/μm) and p=0.417 at 50 Hz (45,V/μm). **(C)** 3D model of the scaled up DEA with reinforcement ribbons. **(D)** comparison of displacement under the same electric field for scaled‐up DEAs with and without reinforcement ribbons. **(E)** displacement and force degradation of scaled‐up DEAs at 1Hz, above 40,V/μm, for 10 h of continuous actuation.

The first stage scans DEA lifetime across operating electric fields and actuation frequencies. The goal is to identify boundary conditions where displacement is high but lifetime is short, making improvement most impactful. At this stage, material choices starts with our best in‐house practices: a carbon black–PDMS mixture is used as the conductive filler, and P3 CNT ink is used as the electrode at a concentration of 2.5 mL/FA (filtration area $FA=50.24,{\mathrm{cm}}^{2}$). Batches of samples are fabricated and tested under a unipolar DC square‐wave actuation (50% duty cycle, rise/fall time ≈ 1.5 ms), which is kept constant across all experiments, across electric fields of 35, 40, 45, and 50 V/μm and frequencies of 1, 5, 10, and 50 Hz. Lifetime (capped at 3 hours) and average displacement are used as evaluation benchmarks. (Figure 3A $\left(i_{d}\right)$ and $\left(i_{t}\right)$). At the lower electric field of 35 V/μm, device performance is stable, with all samples maintaining effective lifetimes beyond 3 hours, although the average displacement remains small. Experiments at this field were stopped after three samples per field–frequency pair due to low variation. At the highest field of 50 V/μm, devices consistently failed early (average lifetime < 1500 s), indicating that this field is outside the practical operating range and too close to electrical breakdown. From these scans, we identified the boundary conditions to be 40 V/μm at 1 Hz and 45 V/μm at 50 Hz. At these conditions, devices exhibit the largest average displacement while still having potential for lifetime improvement.

The next stage focuses on the boundary conditions and scans two material parameters: the electrical connection filler and the CNT electrode concentration. Three filler materials are selected: liquid metal (LM), carbon black–PDMS mixture (CB, default), and carbon grease (CG). Five CNT concentrations are tested (1.8, 2.2, 2.5, 2.9, and 3.3 mL/FA). To maximize sample efficiency, a controlled‐variable approach is used. When scanning filler materials, the CNT concentration is held constant at 2.5 mL/FA, and when scanning CNT concentration, the filler is fixed as CB. Average displacement and lifetime results are shown in Figure 3A‐$ii_{d}$ and ‐$iii_{d}$.

Representative SEM images of rolled DEA cross sections employing different electrical connection fillers are shown in Figure S3. Distinct surface morphologies are observed for the CB and LM fillers, indicating that the choice of conductive material influences the structure of the electrical connection region. These differences may contribute to the distinct lifetime behaviors observed in Figure 3A‐ iii.

At 40 V/μm and 1 Hz, the best‐performing combination is the having CG as the electrical connection filler and 2.5 mL/FA CNT concentration (default). At 45 V/μm and 50 Hz, the highest average displacement is achieved with a CNT concentration of 2.9 mL/FA while using CB as the filler. However, the longest lifetime under the same conditions is obtained using CG as the filler with the default CNT concentration of 2.5 mL/FA. Since the optimal parameters for displacement and lifetime were not tested together, the final stage fabricates devices using the combined parameters of 2.9 mL/FA CNT concentration and CG as the connection filler and tested them at 45 V/μm and 50 Hz. The results show improved performance in both displacement and lifetime.

Through the staged lifetime‐screening workflow, the best material combinations achieved a significant increase in lifetime while also improving the average displacement incrementally compared to the default case. Figure 3B summarizes these comparisons and shows the distribution of measured lifetime and displacement values across independent samples. At 1 Hz and 40 V/μm, the best‐performing configuration uses carbon grease as the electrical connection filler and a CNT concentration of 2.5 mL/FA. Under these conditions, the average lifetime increases by 22% relative to the default baseline and by 72% compared to the worst‐performing case (1.8 mL/FA CNT with CB filler). At 50 Hz and 45 V/μm, the best‐performing combination uses carbon grease as the filler and a CNT concentration of 2.9 mL/FA, resulting in a 99% lifetime increase over the baseline and a 496% increase compared to the worst‐performing configuration. While sample‐to‐sample variation remains present, the improved material combinations consistently shift the measured lifetime toward longer operational durations while maintaining high displacement output.

The present controlled‐variable screening workflow is intended to identify promising operating and material conditions rather than establish a globally optimized actuator design across the full coupled parameter space. Interactions among CNT concentration, conductive filler, actuator geometry, operating conditions, and degradation behavior remain important future directions for more comprehensive optimization studies.

We then transferred the best‐performing parameter combinations identified from the screening study to scaled‐up actuator designs by increasing device length and the number of active layers to enhance force and displacement output for robotic applications. The actuator was enlarged by doubling the active layers from ten to twenty and increasing its length from 1 cm to 2 cm. Two reinforcement ribbons cut from heat‐shrink tubing were wrapped around the rolled DEAs for strain limiting to improve mechanical performance (Figure 3C). The effect of ribbon reinforcement is shown in Figure 3D. Displacement and maximum blocked force were measured across electric fields. The reinforcement does not affect displacement but doubles the maximum blocked force (photographic comparison in Figure S9A. Ten hours of continuous operation above 40 V/μm at 1Hz shows consistent performance with minimal degradation (Figure 3E), maintaining an average axial strain of 5.5% and a specific blocked force of 0.55 N/g. Frequency sweep of the actuator is performed and displacement is measured (Figure S9B). The scaled‐up actuator demonstration therefore serves as a partial validation of the transferability of the screened conditions rather than a fully re‐optimized large‐scale actuator design.

### 2.3 Quadruped Robot Powered by Linear DEAs

A quadruped robot is demonstrated using the scaled‐up linear DEAs. The DEA‐driven robot presented here have the future potential to provide complementary capabilities in specialized operating environments where conventional motor‐driven systems face limitations, rather than replace rigid quadruped robots, which already perform exceptionally well in many application domains [26]. Beyond structural compliance, DEA actuators offer several unique characteristics. DEA actuation is inherently quiet due to the absence of gears and rotating magnetic components. In addition, DEAs are electrostatic actuators with largely non‐metallic structures, potentially enabling operation in environments containing strong magnetic fields where conventional electromagnetic motors may experience interference.

**FIGURE 4:**
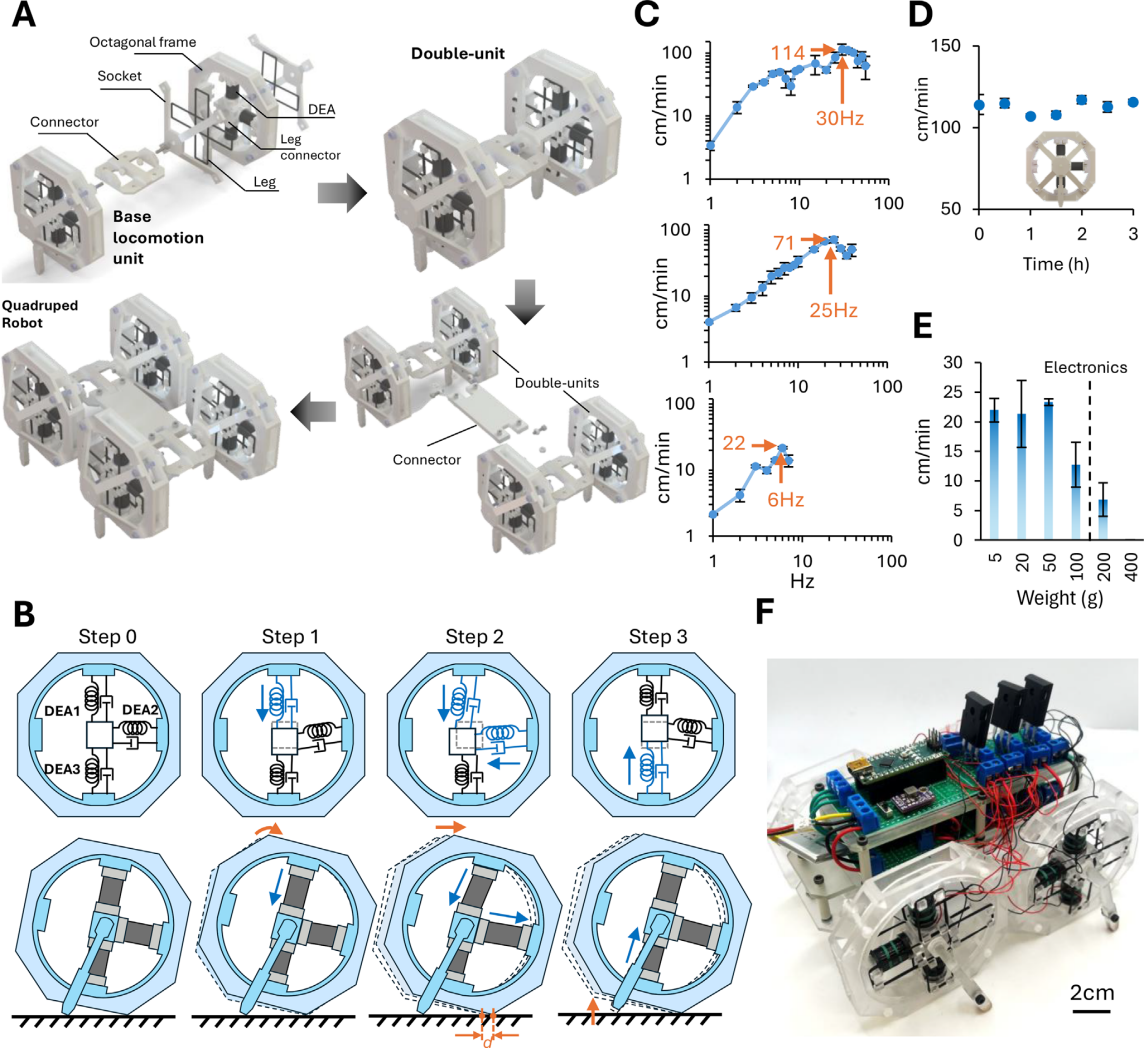
The Modular quadruped walking robot. **(A)** Robot design and assembly. Base locomotion units serve as the building blocks; two units form a double‐unit module, and two double‐units assemble into a quadruped robot. **(B)** Gait mechanism of a single locomotion unit. **(C)** Frequency response of walking speed for 1‐, 2‐, and 4‐unit robot configurations. **(D)** Long‐term speed stability of a base locomotion unit. **(E)** Payload‐dependent walking speed of the tethered quadruped robot. **(F)** Quadruped robot carrying onboard power electronics designed for untethered operation.

Prior DEA‐powered robotic demonstrations have made important progress in lightweight soft robotic systems and untethered small‐scale locomotion [5, 27, 28]. In contrast, the present work demonstrates a scaled‐up DEA‐driven rigid‐frame quadruped capable of sustained walking under externally applied payload loading. This result provides evidence that DEA‐driven locomotion can be successfully scaled to larger robotic platforms. Robots using DEAs as artificial muscles to actuate rigid leg frames have not yet achieved practical, self‐supporting locomotion [29]. The modular walking robot developed here consists of base locomotion units that can be assembled through simple connectors to form larger systems (Figure 4A). When multiple base units are combined, each operates in the same sequence; therefore, regardless of the total number of units, only three high‐voltage switching channels are required. This scalability greatly simplifies the electronics design for more complex robot control.

The base locomotion unit serves as the fundamental building block of the robot platform. Each unit consists of three linear actuators and two legs. Each actuator is connected at one end to a rigid octagonal frame and at the other end to a central leg connector. The current wheel design supports only forward locomotion due to the asymmetric arrangement and actuation sequence of the DEA units. Bidirectional wheel motion may be achievable in future designs through alternative actuator configurations and actuation sequencing strategies. A single unit can move independently, and one complete walking cycle is illustrated in Figure 4B. At the neutral state, the unit is positioned at a slight tilt relative to the ground, with its legs and the front edge of the octagonal frame in contact with the surface. In Step 1, DEA 1 is actuated, driving the leg connector downward to increase friction between the leg and the ground. While DEA 1 remains actuated, DEA 2 is then activated, pushing against the leg connector; the resulting reactive force propels the octagonal frame forward, shifting the unit's center of gravity and producing displacement. In Step 3, DEAs 1 and 2 return to their neutral state, and DEA 3 is actuated to lift and reposition the leg forward, preparing for the next walking cycle. The walking cycle is illustrated in Movie S2, and the analytical modeling of the robot is in Supplementary Text.

Signal frequency has orders of magnitudes influences on walking speed. The frequency response of walking speed was characterized for three robot configurations: a single locomotion unit (single‐unit), two connected locomotion units (double‐unit), and a quadruped robot composed of four units (quadruped bot). All robots were tethered, powered by a high‐voltage benchtop supply (Trek 610E). Video footage of all robot configurations in operation is presented in Movie S3. Actuation sequences were generated through a custom switch board interfacing the actuators with the power supply. The actuation frequency was defined as the inverse of one complete walking cycle. The electric field magnitude was held constant at 40 V/μm. As shown in Figure 4C, the single‐unit robot reached a peak speed of 114 cm/min at 30 Hz, over 100 times faster than its speed at 1 Hz. The peak speed and resonant frequency decreased with larger assemblies: the double‐unit achieved 71 cm/min at 25 Hz, while the quadruped bot reached 22 cm/min at 6 Hz. Frequency sweeps were terminated once each robot ceased to produce measurable displacement. Walking speed comparisons are provided in Movie S4.

The robot demonstrates long‐term operation stability and payload capacity. A base locomotion unit is maintained operating for 3 h at a resonant frequency at 40,V/μm and measured walking speed throughout the time. The walking speed remains constant, implying actuator long‐term stability. The payload carrying capacity of the tethered robot was characterized to guide the design of untethered electronics. A resonant frequency of 6 Hz was selected for the quadruped robot. The robot's speed was recorded under various payloads, as shown in Figure 4E. The tethered quadruped can carry over 200 g, exceeding 100% of its body mass (183 g) and more than 700% of the combined actuator mass (28 g for 12 actuators), while maintaining locomotion. The untethered electronics were designed to weigh between 100 and 200 g (137 g for components and 167 g for the assembled circuit). The circuit diagram is shown in Figure S10. The robot carrying electronics is shown in Figure 4F. However due to the power limitation of the compact high voltage convereters, the electronics cannot sufficiently drives the actuators to achieve locomotion. The system demonstrated here is therefore a prototype, and future work will address this power autonomy challenge.

Several key limitations remain to be addressed for the robot to achieve practical functionality. First, the robot's open‐loop walking trajectory is asymmetric, caused by actuator fabrication inconsistencies and the absence of feedback control. Second, the compact off‐the‐shelf high‐voltage converters currently available (1 W) cannot deliver sufficient power to drive multiple scaled‐up linear actuators, while higher‐power converters are substantially heavier and exceed the robot's payload capacity. Future work will require increasing the robot's load‐carrying capability, either by further scaling up the DEAs or by improving the overall robot architecture. Third, the walking cycle design, actuator configuration, and weight distribution of each locomotion unit can be further optimized to improve speed and reduce performance variation across trials.

## 3 Discussion

In this work, we developed a multi‐channel, multi‐parameter lifetime testing robot for linear DEAs and demonstrated a staged workflow for systematic DEA lifetime screening and robotic integration. Linear DEAs were selected as the initial target system because they are relevant to a wide range of practical applications while their long‐term reliability remains comparatively underexplored. In addition, linear DEAs provide multiple measurable performance metrics, including displacement and blocked force, enabling richer characterization of degradation behavior. Their relatively direct and quantifiable electromechanical response also makes them well suited for systematic lifetime studies and testing‐platform development. We first scanned a parameter space defined by electric field, frequency, electrode material, and electrical connection filler using batch‐fabricated, simplified linear DEAs. The selected parameter combination was then used to scale the actuators in length and layer number, achieving greater force and displacement output. Finally, the scaled‐up DEAs were integrated into a modular quadruped walking robot that demonstrated remarkable payload capacity and long‐term stability.

The demonstrated system automates key testing functions, including programmable actuation, multi‐channel data collection, and electromechanical characterization, but it does not constitute a fully autonomous or closed‐loop self‐driving laboratory. Sample fabrication, parameter selection between experimental stages, and downstream actuator integration remain human‐directed. Therefore, the present work sets an experimental foundation toward device‐level SDL development rather than a completed SDL implementation.

Building from this foundation, a future device‐level SDL would require automated combinatorial fabrication, robotic sample handling, closed‐loop experiment selection, and integrated data‐driven decision‐making. The vision outlined below describes this future direction and separates it from the demonstrated capabilities of the present platform.

### 3.1 Limitation

The limitations of this work lie in hardware reliability, manual sample preparation, and limited data collection throughput. The testing robot is built largely from open‐source electronics, which are generally reliable and cost‐effective, but integration at the system level introduces reliability challenges. For instance, the opto‐couplers experience overheating during extended operation, leading to occasional breakdowns before testing completes; cooling fans were added as a temporary solution. The use of high‐voltage electronics also demands careful circuit design to prevent occasional voltage surges during system startup which can damage components. These reliability issues require periodic manual maintenance, reducing system autonomy and causing sample waste. More broadly, hardware reliability and fault recovery remain common engineering challenges across many SDL systems in materials science and chemistry [30, 31]. Hardware design therefore requires iterative refinement and long‐term system‐level validation.

All DEA samples in this study were manually fabricated. Although 16 devices were made per batch, the process remains time‐consuming. A full batch, including multilayer fabrication and electrical connection, takes roughly eight hours. This constraint limits both the complexity of the parameter space we can explore and the total data volume we can collect. Achieving a fully autonomous system will require automated fabrication with combinatorial capabilities on ingredients.

Data acquisition (DAQ) throughput is another bottleneck. In this work, the DAQ system uses low‐cost chips communicating with the Arduino via the I2C protocol, with data transmitted to a PC through USB. This architecture is reliable and sufficient for current needs, but scaling to a larger number of testing channels or higher sampling rates (>100 Hz per channel) will require advanced DAQ solutions such as FPGA‐based interfaces [32] to handle higher data throughput.

### 3.2 Vision

**FIGURE 5:**
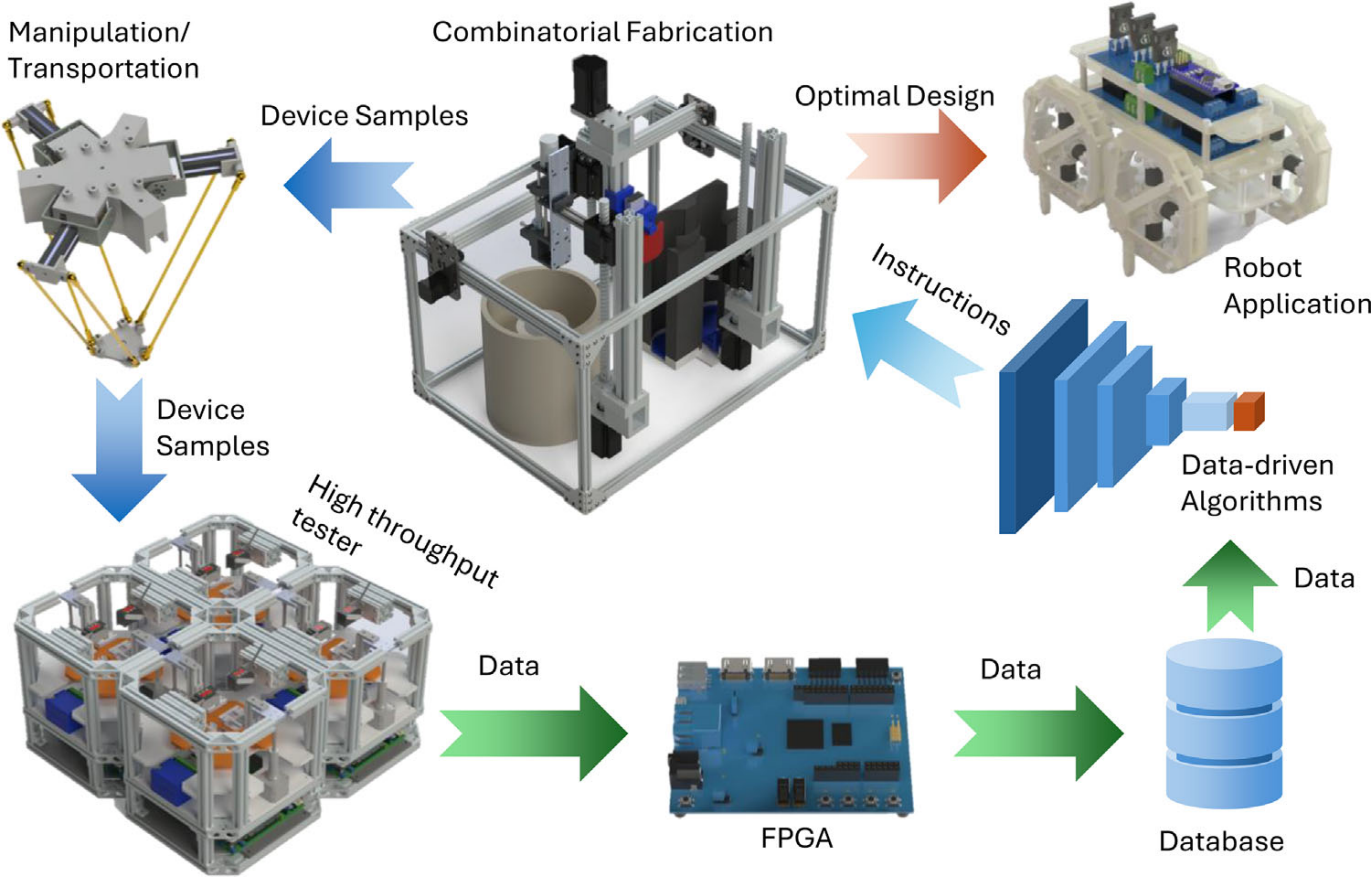
Conceptual vision for a future device‐level SDL workflow for DEA design. (A) combinatorial fabrication robot (under development) produces device samples, which are transferred by a dexterous handling robot to the testing platform. The high‐throughput testing robot conducts comprehensive lifetime measurements, while an FPGA‐based node manages data collection and uploads data to a database. Data‐driven algorithms are used to generate new fabrication parameters for subsequent iterations. The process converges toward an optimized material and device design, which is then transferred for robotic application.

The major challenge in extending SDLs from material‐level to device‐level discovery lies in shifting from single‐variable, static measurements to multi‐variable, time‐domain data acquisition required for reliability testing under varied conditions. Using DEA as a device‐level example, degradation across materials and operating regimes is extremely challenging to be analytically or computationally modeled. Progress on optimization relies on large‐scale experimental data and data‐driven models for performance prediction and rapid screening of design choices. A fully autonomous device‐level SDL must integrate combinatorial fabrication, sample handling, and high‐throughput, multi‐variable testing to close the loop is laid out in Figure 5.

Device‐level SDLs must vary both material composition and fabrication methods. Current systems typically tune one material through a fixed deposition route, whereas devices like DEAs combine electrodes and elastomers with distinct processes. For example, electrodes can be deposited by filter stamping [33], spraying [34], or inkjet printing [35], while elastomers may be applied by spin coating [36], blade coating [10], or 3D printing [37, 38]. Each method leads to different device behavior, requiring the fabrication platforms to adapt to diverse materials and processes. Device assembly and testing require complex and delicate manipulation. In DEAs, multilayer fabrication is followed by cutting, electrical connection, and placement for long‐term testing, all still performed manually. Automation will require dexterous, benchtop‐scale robotic platforms capable of programmable operations for packaging, loading, and transfer between fabrication and testing stages.

Application‐oriented testing demands multi‐channel, multi‐variable setups. For DEAs, meaningful evaluation of force, displacement, and efficiency requires long‐term operation under various waveforms, frequencies, and environmental conditions such as temperature [13], pressure [39], or external load [40]. Battery cyclers provide a good example of a programmable, multi‐channel device tester. However, unlike batteries, which undergo stationary testing, actuators and sensors need dynamic electromechanical measurements, requiring a higher degree of automation and procedure integration. Testing systems must therefore balance throughput with flexibility across applications.

The present platform establishes the data‐collection and testing infrastructure required for future data‐driven and closed‐loop DEA design workflows. The collected data include discrete fabrication and operating parameters, such as material selection, CNT concentration, filler type, electric field, and actuation frequency, together with corresponding performance metrics including lifetime, average blocked force, average displacement, and impedance. In addition, the platform acquires time‐domain signals from displacement, blocked‐force, and current‐feedback measurements throughout device operation.

These datasets could support several future machine‐learning‐assisted DEA design approaches. First, the time‐domain electrical and mechanical data could be used to train predictive models for early lifetime estimation, where short‐duration actuation data are used to predict long‐term operational lifetime and degradation behavior. Similar approaches have previously been explored in both DEA energy‐efficiency prediction [16] and battery cycle‐life prediction research [21]. Second, supervised learning models could be trained to map fabrication and operating parameters to device‐level performance metrics such as lifetime, displacement, or blocked force. Such models could help solve the device inverse‐design problem by finding material and operating conditions given performance targets. Third, Bayesian‐guided experiment‐selection strategies could be integrated with these predictive models to iteratively refine fabrication and operating parameters to converge to the targeted performance. The present high‐throughput testing platform and structured dataset already provide the foundation for training models for device rapid‐evaluation and inverse design. However, realizing fully closed‐loop iterative improvement and autonomous experiment selection would require realizing the envisioned fully automated system in Figure 5.

## 4 Experimental Section

### 4.1 DEA Materials and Fabrication

Linear DEAs are fabricated by first producing a multilayer DEA sheet, trimming excess elastomer to expose an edge for electrical connection, then rolling the sheet and encapsulating it between two rigid caps (Figure S2). The cap assembly is designed to maximize the effective conductive area. An adhesive is used to attach an ultra‐flex copper wire to a circular conductive metallic sheet (aluminum or copper), increasing the contact surface. A conductive filler material is applied to the exposed elastomer–electrode interface and serves as the colloidal layer between the elastomer and the metallic sheet. The multilayer fabrication procedures follow established methods [33, 41]. The caps are 3D printed using a Form 4 resin printer (Formlabs). Elastosil P7670 (Wacker Chemie AG) is selected as the elastomer, and CNT ink is prepared in‐house using P3 CNT powder (Carbon Solutions, Inc.), following the published procedure [9]. Ink consistency is verified by measuring optical transmittance and adjusting the density accordingly.

### 4.2 Conductive Filler

Three conductive filler materials are examined in this work: carbon grease (CG), eutectic gallium–indium liquid metal (LM) [42], and a PDMS–carbon black mixture (CB) [43]. Carbon grease is an off‐the‐shelf product (MG Chemicals), while LM and CB are prepared in‐house. The procedure for preparing LM is as follows: a 1:2 weight ratio of indium to eutectic gallium is heated to the liquid state in a 70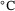 water bath for 1 h, then mixed on a hot plate at 60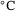 for 12 h or until uniform. The procedure for preparing CB is as follows: the mixture is formulated using a 100:3 weight ratio of PDMS (part B) to carbon black powder as the conductive medium. Ingredients are mixed in a two‐axis centrifugal mixer (FlackTek) at 2500 RPM for 2 min, with fractional additions of carbon black until the target concentration is reached. Multiple spin cycles ensure even particle distribution throughout the mixture.

### 4.3 Measurement of DEA Properties

Displacement of the DEAs is measured using a laser displacement sensor (Panasonic HGC‐1030 series). A small piece of white paper is attached to the top surface of each DEA to provide a stable reflective surface. Blocked force is measured using FSG005WNPB force sensors (Honeywell). These sensors are mounted on linear actuators that automatically clamp onto the DEA samples to restrict motion. The clamping pressure is maintained using force‐feedback from the sensors themselves, with a bias force of 0.6 N for DEA testing samples and 1 N for scaled‐up actuators. Operation of the linear actuators is shown in Movie S1. Current and voltage feedback are obtained directly from the monitoring channels of the high‐voltage converters (XP Power HRC series). Capacitance is measured using the impedance meter function of the Analog Discovery 3 (Digilent), with each sweep performed from 1 kHz to 1 MHz. Meaured capacitance prior and after long‐term actuation for some DEA samples is ploted in Figure S7. Higher actuation voltages lead to greater capacitance degradation, while the influence of actuation frequency on degradation is less obvious.

### 4.4 Programmable High Voltage Power Supply

The dual‐channel high‐voltage power supply is built around programmable high‐voltage converters. The circuit is modular and can accommodate either XP Power HRC‐series 5W converters or HRL30‐series 30W converters, depending on the power requirements of the DEAs (5W is sufficient for the Elastosil‐based actuators used in this work). The circuit diagram is shown in Figure S5. This modular design enables straightforward scalability; additional converter channels can be added by connecting to the shared power line and linking their programming and feedback channels to the MCU. High‐frequency square‐wave actuation is generated using a dual opto‐coupler configuration (HVM OR‐100 series), where one opto‐coupler manages charging, and the other manages discharging, operating in a complementary flip–flop manner so that only one is active at a time.

### 4.5 Walking Robot

The rigid framework components of the walking robot are printed using a Form 4 printer with standard clear SLA resin. All rigid parts are assembled using nylon or metallic screws. DEAs are mounted to the frame using double‐sided tape. Sandpaper can be attached to the bottom of the legs to increase ground friction, and weight distribution can be adjusted in a modular fashion by attaching small magnets to the metallic screws.

Long‐term speed tracking of the tethered quadruped robot is challenging due to cable length constraints and the lack of closed‐loop control to maintain continuous walking. To assess long‐term performance, we alternate between two measurement modes: allowing the robot to walk freely over a fixed distance to record ground speed, and blocking its motion with an obstacle while it continues actuating so that the actuators accumulate operating time. Repeating these two modes over a three‐hour window provides an effective estimate of long‐term speed trends while accommodating the limitations of a tethered setup.

## Author Contributions

A.L. and M.D. initiated the research concept. A.L. built the testing robot. A.L., S.S., V.J‐S., fabricated the DEAs. A.L. performed data collection and processing. A.W. designed the programmable power supply. A.Y. developed the walking cycle and analytical modeling for the walking robot. A.L. built and tested the walking robot. M.F. conducted material characterization experiments. V.R. helped with figure generation and vision development. C.T. made and tested electrical filler materials. A.L. wrote the paper. M.D. edited the paper.

## Funding

Natural Sciences and Engineering Resesarch Council of Canada ‐ Discovery Grant RGPIN‐2021‐02791. AFRL MidAtlantic Hub ‐ Prototyping Award. University of Connecticut ‐ SURF Award Summer 2025 (M. Francoeur). Naval Institute for Undersea Vehicle Technologies ‐ SEED 61. NASA Connecticut Graduate Fellowships (A. White, S. Sandhu, and V. Jimenez‐Santiago).

## Conflicts of Interest

The authors declare no conflicts of interest.

## Supporting information


**Supporting File 1**: advs77008‐sup‐0001‐SuppMat.pdf.

**Supporting File 2**: advs77008‐sup‐0002‐MovieS1‐S4.zip.

## Data Availability

The data that support the findings of this study are available from the corresponding author upon reasonable request.

## References

[1] Y. Chen , H. Zhao , J. Mao et al., “Controlled Flight of a Microrobot Powered by Soft Artificial Muscles,” Nature 575, no. 7782 (2019): 324–329. 31686057 10.1038/s41586-019-1737-7

[2] J. Shintake , S. Rosset , B. E. Schubert , D. Floreano , and H. R. Shea , “A Foldable Antagonistic Actuator,” IEEE/ASME Transactions on Mechatronics 20, no. 5 (2014): 1997–2008.

[3] F. Berlinger , M. Duduta , H. Gloria , D. Clarke , R. Nagpal , and R. Wood , “A Modular Dielectric Elastomer Actuator to Drive Miniature Autonomous Underwater Vehicles,” in 2018 IEEE International Conference on Robotics and Automation (ICRA) (IEEE, 2018), 3429–3435.

[4] D. Flores , S. Sandhu , A. White et al., “RoboNautilus: A Cephalopod‐Inspired Soft Robotic Siphon for Underwater Propulsion,” npj Robotics 3, no. 1 (2025): 17.

[5] X. Ji , X. Liu , V. Cacucciolo et al., “An Autonomous Untethered Fast Soft Robotic Insect Driven by Low‐Voltage Dielectric Elastomer Actuators,” Science Robotics 4, no. 37 (2019): eaaz6451. 33137720 10.1126/scirobotics.aaz6451

[6] Q. Pei , M. A. Rosenthal , R. Pelrine , S. Stanford , and R. D. Kornbluh , “Multifunctional Electroelastomer Roll Actuators and Their Application for Biomimetic Walking Robots,” in Smart Structures and Materials 2003: Electroactive Polymer Actuators and Devices (EAPAD), Vol 5051 (SPIE, 2003), 281–290.

[7] C. Tang , B. Du , S. Jiang et al., “A Pipeline Inspection Robot for Navigating Tubular Environments in the Sub‐Centimeter Scale,” Science Robotics 7, no. 66 (2022): eabm8597. 35613300 10.1126/scirobotics.abm8597

[8] S. Sandhu , A. L. Li , C. Tugui , and M. Duduta , “Miniature Dielectric Elastomer Actuator Probe Inspecting Confined Spaces Embedding a CMOS Sensor,” in 2025 IEEE International Conference on Robotics and Automation (ICRA) (IEEE, 2025), 6963–6968.

[9] E. Hajiesmaili and D. R. Clarke , “Dielectric Elastomer Actuators,” Journal of Applied Physics 129, no. 15 (2021): 151102.

[10] S. Jiang , C. Tang , X.‐J. Liu , and H. Zhao , “Long‐Life‐Cycle and Damage‐Recovery Artificial Muscles via Controllable and Observable Self‐Clearing Process,” Advanced Engineering Materials 24, no. 4 (2022): 2101017.

[11] D. Kühnel , F. B. Albuquerque , V. Py , and H. Shea , “Automated Test Setup to Quantify the Lifetime of Dielectric Elastomer Actuators Under a Wide Range of Operating Conditions,” Smart Materials and Structures 30, no. 6 (2021): 065020.

[12] M. Matysek , P. Lotz , and H. F. Schlaak , “Lifetime Investigation of Dielectric Elastomer Stack Actuators,” IEEE Transactions on Dielectrics and Electrical Insulation 18, no. 1 (2011): 89–96.

[13] F. B. Albuquerque and H. Shea , “Influence of Electric Field, Temperature, Humidity, Elastomer Material, and Encapsulation on the Lifetime of Dielectric Elastomer Actuators (DEAs) Under DC Actuation,” Smart Materials and Structures 30, no. 12 (2021): 125022.

[14] D. Bruch , “Multifunctional Test Rig for Accelerated Assessment of Electromechanical Characteristics and Failure Mechanisms of Dielectric Elastomer Transducers,” Saarländische Universitäts‐und Landesbibliothek (2024).

[15] A. L. Li , S. Lee , H. Shahsa , and M. Duduta , “Real‐Time High‐Voltage Capacitance for Rapid Evaluation of Dielectric Elastomer Actuators,” Soft Matter 18, no. 37 (2022): 7123–7130. 36082902 10.1039/d2sm00690a

[16] A. Li , P. Cuvin , S. Lee , J. Gu , C. Tugui , and M. Duduta , “Data‐Driven Long‐Term Energy Efficiency Prediction of Dielectric Elastomer Artificial Muscles,” Advanced Functional Materials 34, no. 42 (2024): 2406710.

[17] D. P. Tabor , L. M. Roch , S. K. Saikin et al., “Accelerating the Discovery of Materials for Clean Energy in the Era of Smart Automation,” Nature Reviews Materials 3, no. 5 (2018): 5–20.

[18] B. Li , B. Deng , W. Shou et al., “Computational Discovery of Microstructured Composites with Optimal Stiffness‐Toughness Trade‐Offs,” Science Advances 10, no. 5 (2024): eadk4284. 38306429 10.1126/sciadv.adk4284 PMC10836719

[19] B. P. MacLeod , F. G. Parlane , T. D. Morrissey et al., “Self‐Driving Laboratory for Accelerated Discovery of Thin‐Film Materials,” Science Advances 6, no. 20 (2020): eaaz8867. 32426501 10.1126/sciadv.aaz8867 PMC7220369

[20] A. E. Siemenn , B. Das , K. Ji , F. Sheng , and T. Buonassisi , “A Self‐Supervised Robotic System for Autonomous Contact‐Based Spatial Mapping of Semiconductor Properties,” Science Advances 11, no. 27 (2025): eadw7071. 40614181 10.1126/sciadv.adw7071 PMC12227050

[21] K. A. Severson , P. M. Attia , N. Jin et al., “Data‐Driven Prediction of Battery Cycle Life Before Capacity Degradation,” Nature Energy 4, no. 5 (2019): 383–391.

[22] J. Ulrich , M. Stefanec , F. Rekabi‐Bana et al., “Autonomous Tracking of Honey Bee Behaviors Over Long‐Term Periods with Cooperating Robots,” Science Robotics 9, no. 95 (2024): eadn6848. 39413166 10.1126/scirobotics.adn6848

[23] Z. Ren , S. Kim , X. Ji et al., “A High‐Lift Micro‐Aerial Robot Powered by Low‐Voltage and Long‐Endurance Dielectric Elastomer Actuators,” Advanced Materials 34, no. 7 (2022): 2106757. 10.1002/adma.202106757 34839551

[24] X. Han , M. Ouyang , L. Lu , J. Li , Y. Zheng , and Z. Li , “A Comparative Study of Commercial Lithium‐Ion Battery Cycle Life in Electrical Vehicle: Aging Mechanism Identification,” Journal of Power Sources 251 (2014): 38–54.

[25] F. Carpi , I. Anderson , S. Bauer et al., “Standards for Dielectric Elastomer Transducers,” Smart Materials and Structures 24, no. 10 (2015): 105025.

[26] Y. Fan , Z. Pei , C. Wang , M. Li , Z. Tang , and Q. Liu , “A Review of Quadruped Robots: Structure, Control, and Autonomous Motion,” Advanced Intelligent Systems 6, no. 6 (2024): 2300783.

[27] Q. Pei , M. Rosenthal , S. Stanford , H. Prahlad , and R. Pelrine , “Multiple‐Degrees‐of‐Freedom Electroelastomer Roll Actuators,” Smart Materials and Structures 13, no. 5 (2004): N86.

[28] C. T. Nguyen , H. Phung , P. T. Hoang , T. D. Nguyen , H. Jung , and H. R. Choi , “Development of an Insect‐Inspired Hexapod Robot Actuated by Soft Actuators,” Journal of Mechanisms and Robotics 10, no. 6 (2018): 061016.

[29] C. T. Nguyen , H. Phung , T. D. Nguyen et al., “A Small Biomimetic Quadruped Robot Driven by Multistacked Dielectric Elastomer Actuators,” Smart Materials and Structures 23, no. 6 (2014): 065005.

[30] S. Musslick , L. K. Bartlett , S. H. Chandramouli et al., “Automating the Practice of Science: Opportunities, Challenges, and Implications,” Proceedings of the National Academy of Sciences 122, no. 5 (2025): e2401238121. 10.1073/pnas.2401238121 PMC11804648 39869810

[31] S. X. Leong , C. E. Griesbach , R. Zhang et al., “Steering Towards Safe Self‐Driving Laboratories,” Nature Reviews Chemistry 9, no. 10 (2025): 707–722. 40826175 10.1038/s41570-025-00747-x

[32] G. R. D. Prabhu and P. L. Urban , “Elevating Chemistry Research with a Modern Electronics Toolkit,” Chemical Reviews 120, no. 17 (2020): 9482–9553. 32786428 10.1021/acs.chemrev.0c00206

[33] M. Duduta , R. J. Wood , and D. R. Clarke , “Multilayer Dielectric Elastomers for Fast, Programmable Actuation Without Prestretch,” Advanced Materials 28, no. 36 (2016): 8058–8063. 27376638 10.1002/adma.201601842

[34] Z. Peng , Y. Shi , N. Chen , Y. Li , and Q. Pei , “Stable and High‐Strain Dielectric Elastomer Actuators Based on a Carbon Nanotube‐Polymer Bilayer Electrode,” Advanced Functional Materials 31, no. 9 (2021): 2008321.

[35] C. Baechler , S. Gardin , H. Abuhimd , and G. Kovacs , “Inkjet Printed Multiwall Carbon Nanotube Electrodes for Dielectric Elastomer Actuators,” Smart Materials and Structures 25, no. 5 (2016): 055009.

[36] P. Lotz , M. Matysek , and H. F. Schlaak , “Fabrication and Application of Miniaturized Dielectric Elastomer Stack Actuators,” IEEE/ASME Transactions on Mechatronics 16, no. 1 (2010): 58–66.

[37] A. Chortos , E. Hajiesmaili , J. Morales , D. R. Clarke , and J. A. Lewis , “3D Printing of Interdigitated Dielectric Elastomer Actuators,” Advanced Functional Materials 30, no. 1 (2020): 1907375.

[38] C. Tugui , M. Cazacu , D. M. Manoli , A. Stefan , and M. Duduta , “All‐Silicone 3D Printing Technology: Toward Highly Elastic Dielectric Elastomers and Complex Structures,” ACS Applied Polymer Materials 5, no. 10 (2023): 7936–7946.

[39] G. Li , X. Chen , F. Zhou et al., “Self‐Powered Soft Robot in the Mariana Trench,” Nature 591, no. 7848 (2021): 66–71. 33658693 10.1038/s41586-020-03153-z

[40] M. Duduta , E. Hajiesmaili , H. Zhao , R. J. Wood , and D. R. Clarke , “Realizing the Potential of Dielectric Elastomer Artificial Muscles,” Proceedings of the National Academy of Sciences of the United States of America 116, no. 7 (2019): 2476–2481. 30679271 10.1073/pnas.1815053116 PMC6377461

[41] H. Zhao , A. M. Hussain , M. Duduta , D. M. Vogt , R. J. Wood , and D. R. Clarke , “Compact Dielectric Elastomer Linear Actuators,” Advanced Functional Materials 28, no. 42 (2018): 1804328.

[42] M. R. Khan , C. B. Eaker , E. F. Bowden , and M. D. Dickey , “Giant and Switchable Surface Activity of Liquid Metal via Surface Oxidation,” Proceedings of the National Academy of Sciences of the United States of America 111, no. 39 (2014): 14047–14051. 25228767 10.1073/pnas.1412227111 PMC4191764

[43] S. Rosset and H. R. Shea , “Flexible and Stretchable Electrodes for Dielectric Elastomer Actuators,” Applied Physics A 110 (2013): 281–307.

