# Supplementary material for: A Robotic Testing Platform for Pipelined Discovery of Resilient Dielectric Elastomer Actuators
Source: Adv Sci (Weinh). 2026 Aug 11:e77008. Online ahead of print. doi: 10.1002/advs.77008 (PMC13460422; doi:10.1002/advs.77008)
Supplement: Supplementary file 1 [file ADVS-9999-e77008-s001.pdf]

Supplementary materials

Supplementary Text

Figs. S1 to S7

Movies S1 to S4

Supplementary Materials for
A Robotic Testing Platform for Pipelined Discovery of Resilient Soft Actuators

Ang (Leo) Li*, Alexander Yin, Alexander White, Sahib Sandhu, Anatol Gogoj,
Denise Maria de Andrade, Matthew Francoeur, Van Remenar, Codrin Tugui, Mihai Duduta*
*Corresponding author. Email: shffleo.li@mail.utoronto.ca , mihai.duduta@uconn.edu

This PDF file includes:

Supplementary Text
Figures S1 to S7
Captions for Movies S1 to S4

Other Supplementary Materials for this manuscript:

Movies S1 to S4

Supplementary Text

Analytical modeling of a base locomotion unit robot

To gain a better understanding of how the a singular wheel moves, a simplified model was created to simulate the leg's motion and subsequently the amount of force it can generate due to the deformation of the attached DEAs. The single wheel has an outer octagon frame that has three DEAs attached to a central block connected to a leg. Activating the DEAs cause the central block and its attached leg to move. This motion causes the wheel to move as it shifts the center of gravity and exerts force through the leg that is touching the floor. The length of the leg and the angle at which it is attached to the frame will affect how the wheel is positioned with respect to the floor. The leg is designed to extend slightly outside the frame, causing the wheel to be positioned at a slight angle with respect to the floor. In this configuration, the wheel will only have two points of contact with the floor, the leg and a corner of the frame, shown in **Figure 4B**. As the DEAs are powered, the position of the leg will shift causing the body to change its angle with the floor. The center block's location can be defined using its relation to the frame. Its height compared to the bottom of the frame is defined at h_c and its horizontal distance from the pivot corner is defined as w_c both in centimeters. Its location will change depending on which DEA is active as indicated by e_n which defines the linear elongation for the given DEA, n. To simplify the simulation, all DEAs are assumed to be ideal such that elongation, e_n and force F_n are the maximum recorded values from testing. Additionally, it is assumed that they are not antagonistic to one another even though they are all attached to the center block because they are flexible and the elongation is small. This results in equations 1 and 2.

$$h_c = \frac{h}{2} - e_1 + e_3 \tag{S1}$$

$$w_c = \frac{b}{2} + e_2 \tag{S2}$$

Using the central block's position and the leg's configuration, further information about how the leg is positioned with respect to the frame can be calculated. The length of the leg l and the attached angle θ_l can be used to calculate the vertical length between the end of the leg and the bottom of the frame, δ_h shown in equation 3. This information can then be used to calculate the

length of the leg that is outside the frame, δ_l, shown in equation 4. This is then used to find the horizontal distance between the pivot point and the point the leg crosses over the base, δ_w shown in equation 5.

$$\delta_h = l\cos(\theta_l) - h_c \tag{S3}$$

$$\delta_l = \delta_h \sec(\theta_l) \tag{S4}$$

$$\delta_w = w_c + (l - \delta_l)\sin(\theta_l) \tag{S5}$$

With the additional information, the distance between the two contact points on the floor, d, can be calculated, as shown in figure 5. This can then be used to find the tilt the body is compared to the ground denoted as θ_b and shown in equation 7.

$$d = \sqrt{\delta_l^2 + \delta_w^2 - 2\delta_l\delta_w\cos\left(\theta_l + 90\right)} \tag{S6}$$

$$\theta_b = \arcsin\left(\frac{\delta_l\sin(\theta_l + 90)}{d}\right) \tag{S7}$$

The body angle is then used to understand the internal forces that are created by the attached DEAs. The forces induced by the DEAs change depending on the posture of the frame. For further simplification, the forces are split into its vertical F_y and horizontal F_x components shown in equations 8 and 9.

$$F_x = (-F_1 + F_3) * \sin(\theta_b) - F_2\cos(\theta_b) \tag{S8}$$

$$F_y = (-F_1 + F_3) * \cos(\theta_b) - F_2\cos(\theta_b) \tag{S9}$$

With the simulation, different leg configurations, DEA specifications, and pulse timings can be explored to further improve the wheel's performance. Currently, a simple 3 step open loop control is used for a single wheel shown in the **movie s1**. More complex controls involving different pulse timings such as a descending ramp to turn off the DEA could prove useful to the wheels performance.

	This work	MAPLE platform [1]	M. Matysek et al. [2]	S. Jiang et al. [3]	D. Bruch et al. [4]
**Platform capabilities**					
Multi-channel testing	✓	✓	✓		✓
Programmable voltage waveform	✓	✓		✓	✓
Displacement measurement	✓	✓	✓	✓	✓
Blocked-force measurement	✓		✓		✓
Impedance/electrical property measurement	✓			✓	✓
Automated mode switching	✓				
**Experimental scope**					
Long-term degradation monitoring	✓	✓	✓	✓	✓
Frequency /electrical field scanning	✓			✓	✓
Material parameters scanning	✓				
Environmental condition testing		✓			✓
**Design consideration**					
Benchtop profile	✓	✓		✓	
Open-source electronics	✓				
Modular channel scalability	✓	✓			✓
**Research conducted**					
Testing-guided actuator design	✓				
Integration to application	✓				

Figure S1: **Comparison table of DEA testing platforms** Comparison of the proposed DEA testing platform with existing DEA testing systems, including the MAPLE platform (*1*), Matysek et al. (*2*), Jiang et al. (*3*), and Bruch et al. (*4*). The comparison highlights differences in platform capabilities, experimental scope, system design considerations, and demonstrated integration toward actuator optimization and robotic application.

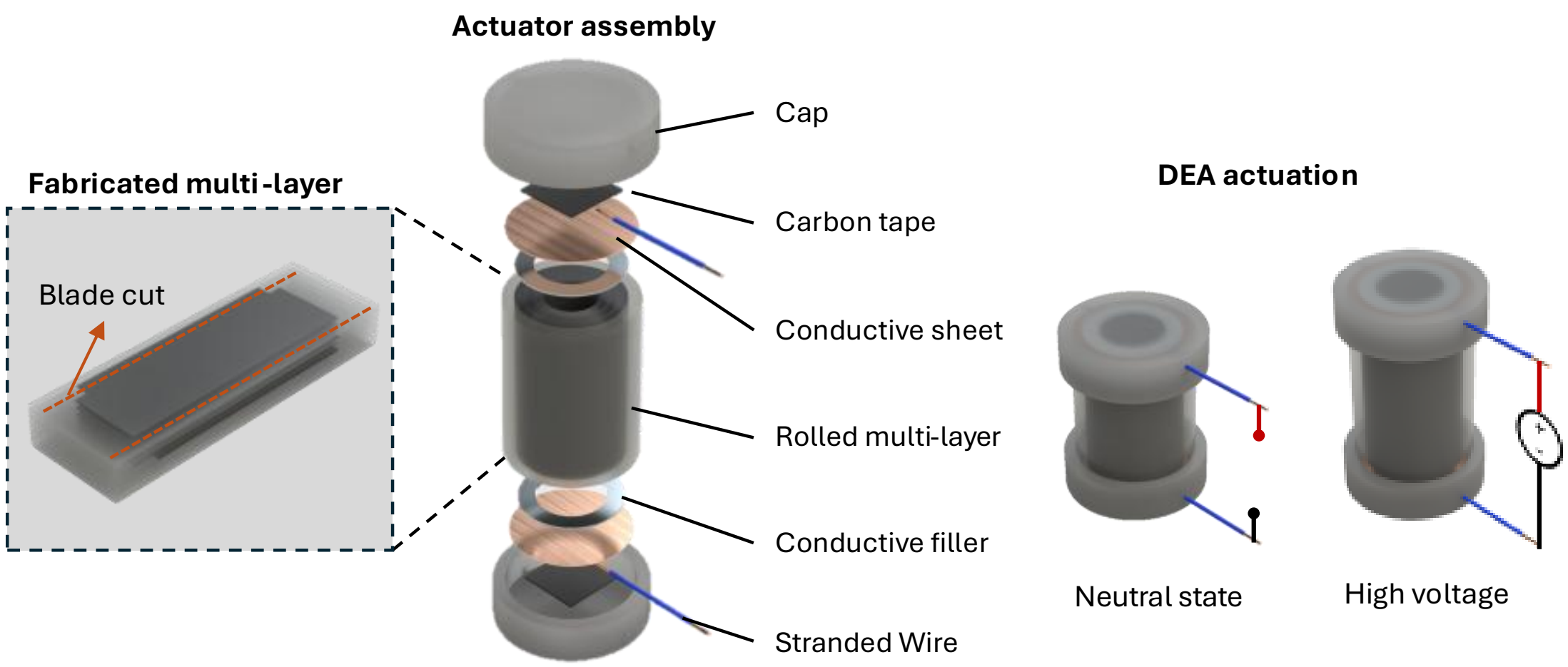

Figure S2: **Linear actuator assembly.** The multilayer actuator is fabricated using standard procedures well-documented in literature (*5, 6*). After trimming, the multilayers are rolled and placed between two end caps. Carbon tape provides an adhesive interface for conductors, connecting stranded copper wires and conductive sheets. The wires serve as external electrical leads, while the conductive sheets extend the effective contact area with the DEA. A conductive filler layer is applied between the conductive sheet and the multilayer cross section; this layer is critical to the device's cycle-life performance and is a key focus of material selection in this work. The actuator expands linearly along its length when an electric field is applied across its terminals.

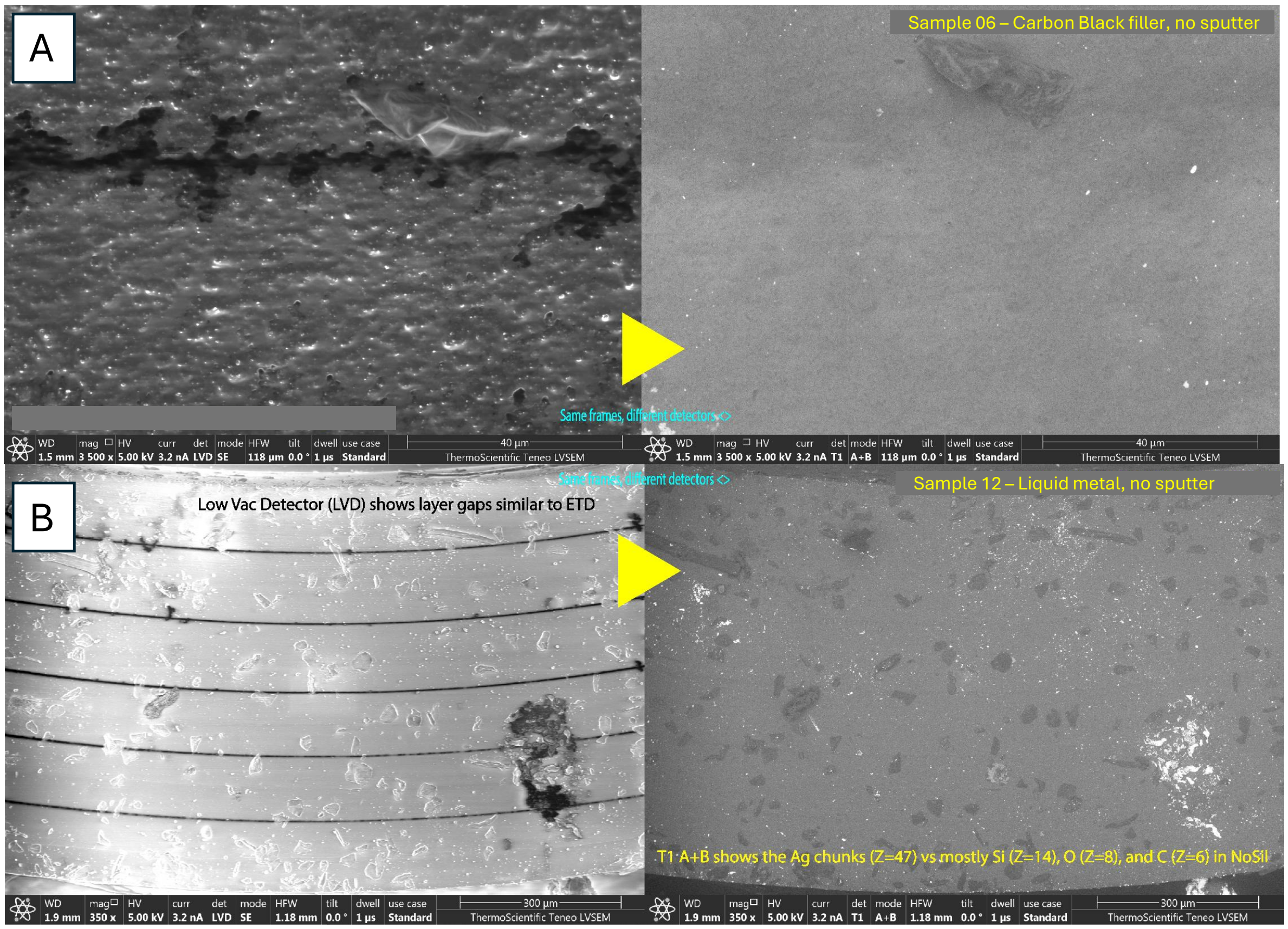

Figure S3: **SEM characterization of rolled DEA cross sections employing different electrical connection fillers.** (A) Device using a carbon-black/PDMS (CB) filler. (B) Device using a liquid-metal (LM) filler. For each sample, the left image was acquired using the low-vacuum detector (LVD), highlighting surface topography, while the right image was acquired using the T1 backscatter detector, providing compositional contrast. Distinct morphologies are observed in the electrical connection region for the two filler materials, indicating that the conductive filler influences the interfacial structure of the rolled actuator.

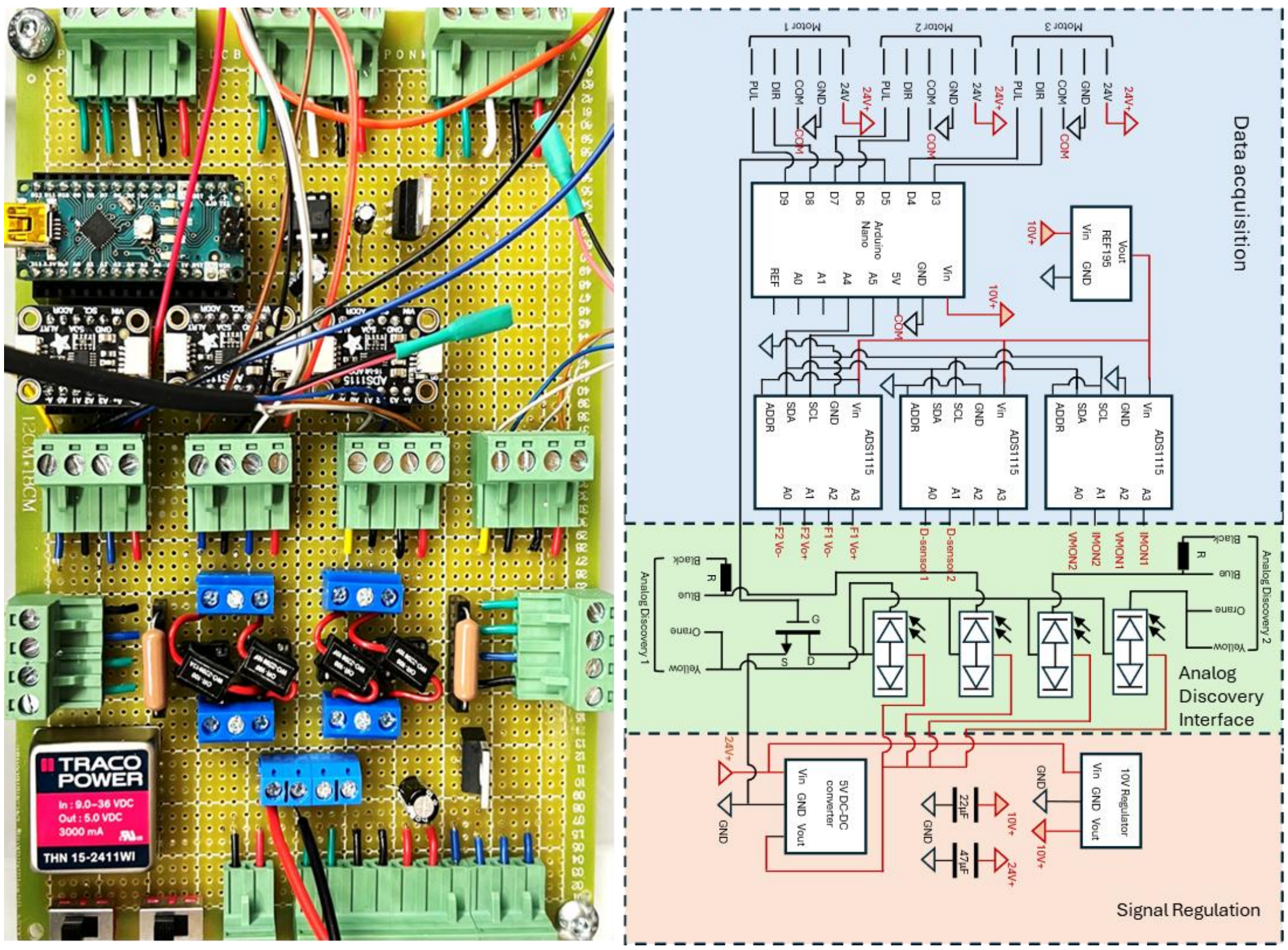

Figure S4: **Data acquisition circuit for the testing robot.** (Left) the photo of the circuit board. (Right) The circuit topology.

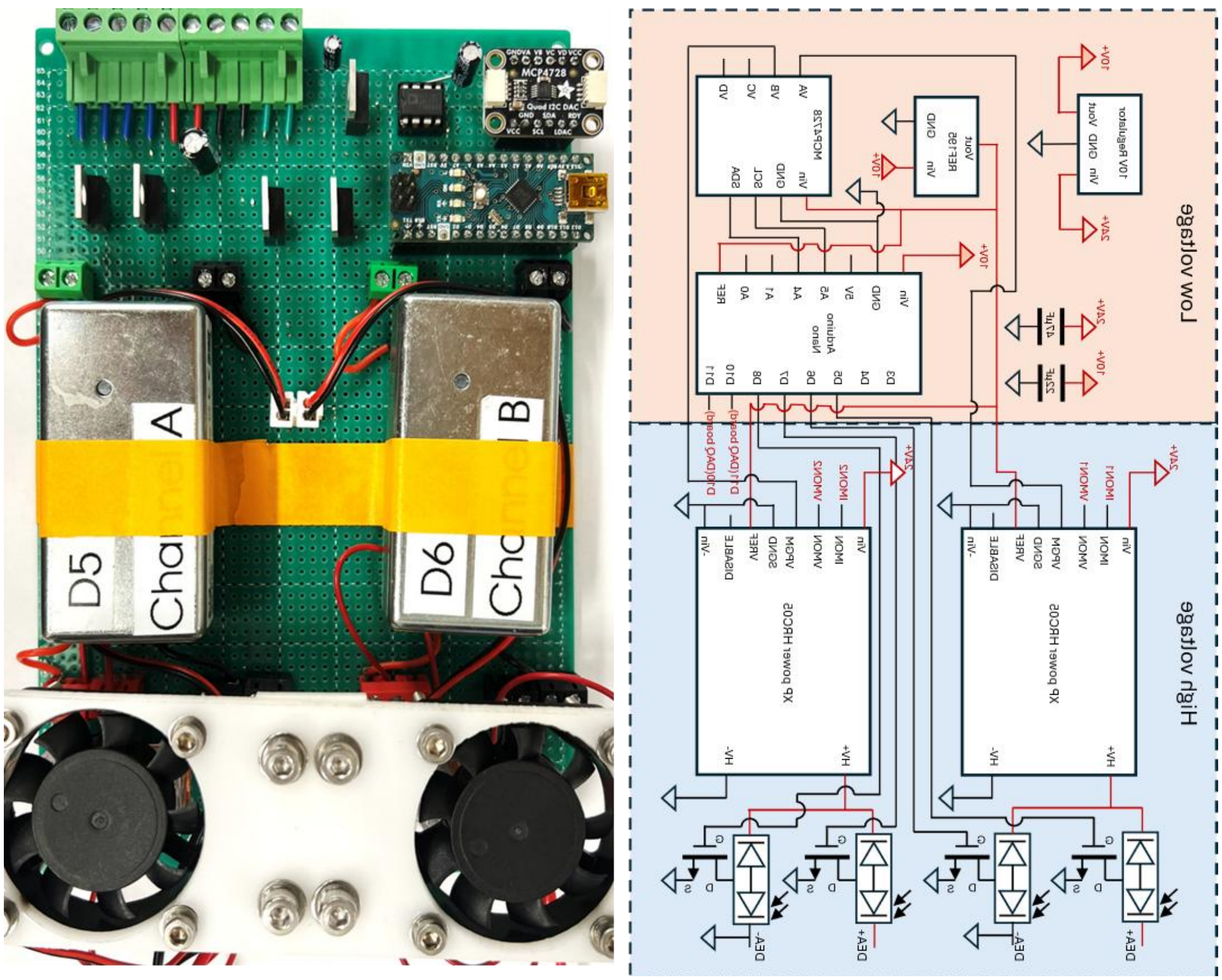

Figure S5: **Programmable power supply circuit for the testing robot.** (Left) the photo of the circuit board. (Right) The circuit topology (cooling fans are not included).

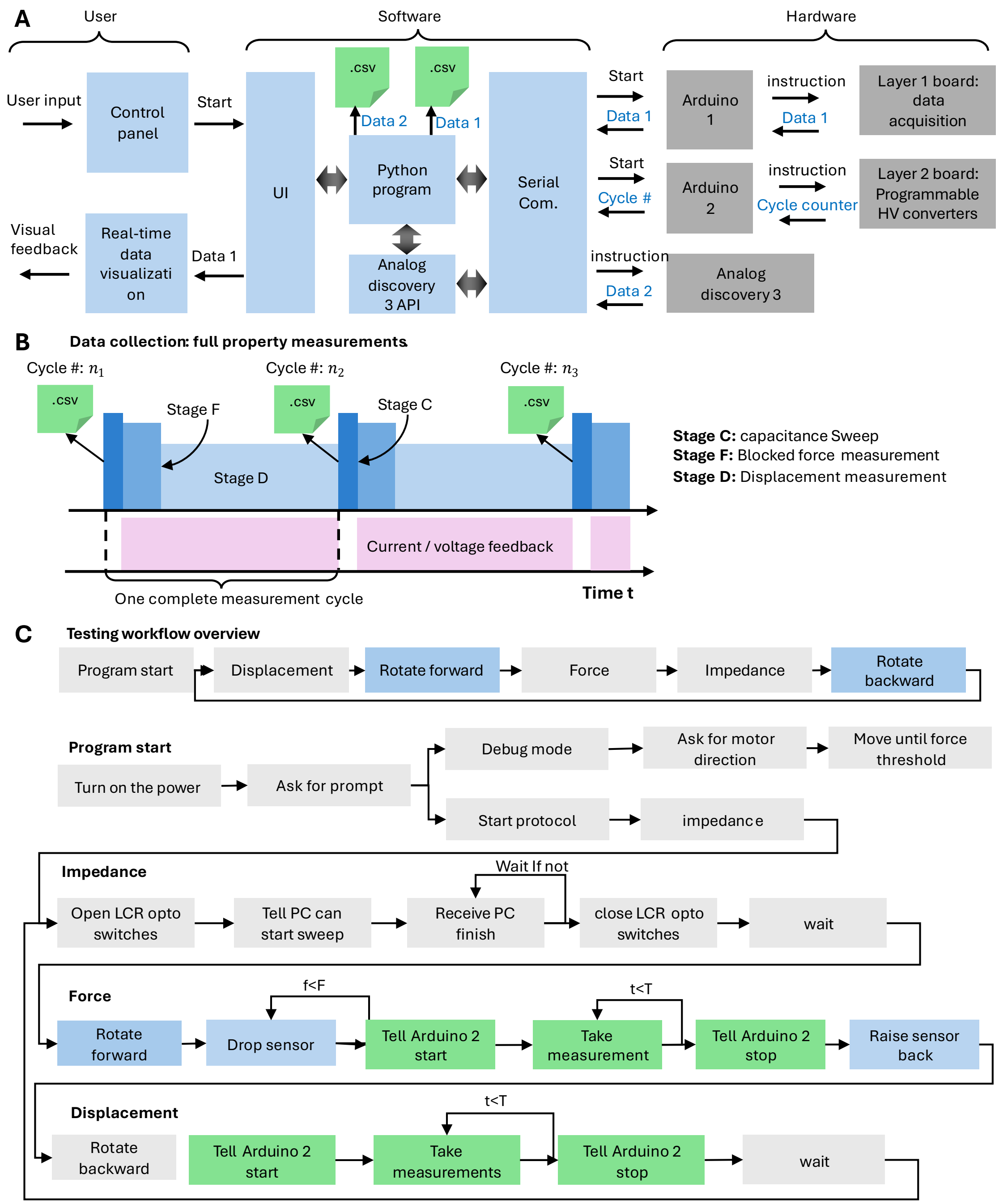

Figure S6: **Testing robot architecture and workflow.** (A) System architecture from user interface to hardware. (B) Data collection sequence for full electromechanical measurements. The robot periodically switches measurement modes to capture complete datasets; however, most data in this work were collected using simplified protocols measuring either force or displacement. (C) Workflow of Arduino 1 (top-layer control and DAQ board). Motor actions are shown in blue, and Arduino 2 (high-voltage board) actions in green.

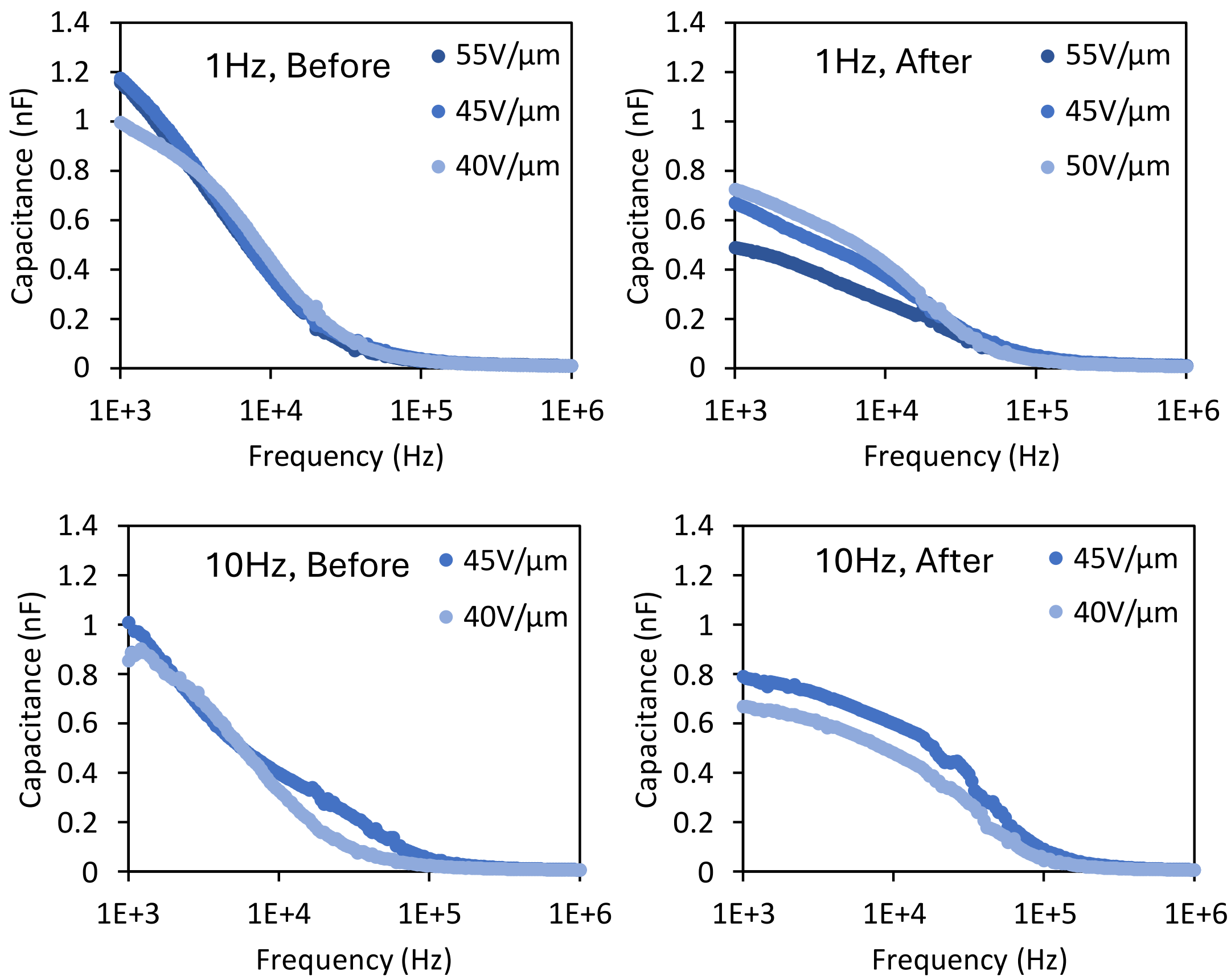

Figure S7: **Capacitance–frequency sweep measurements of device samples under different electric fields and actuation frequencies.** Measurements were performed using Analog Discovery 3 modules mounted on the testing robot. The left plots show capacitance before testing, and the right plots show capacitance after testing. Higher actuation voltages lead to greater capacitance degradation, while the influence of actuation frequency on degradation is less pronounced.

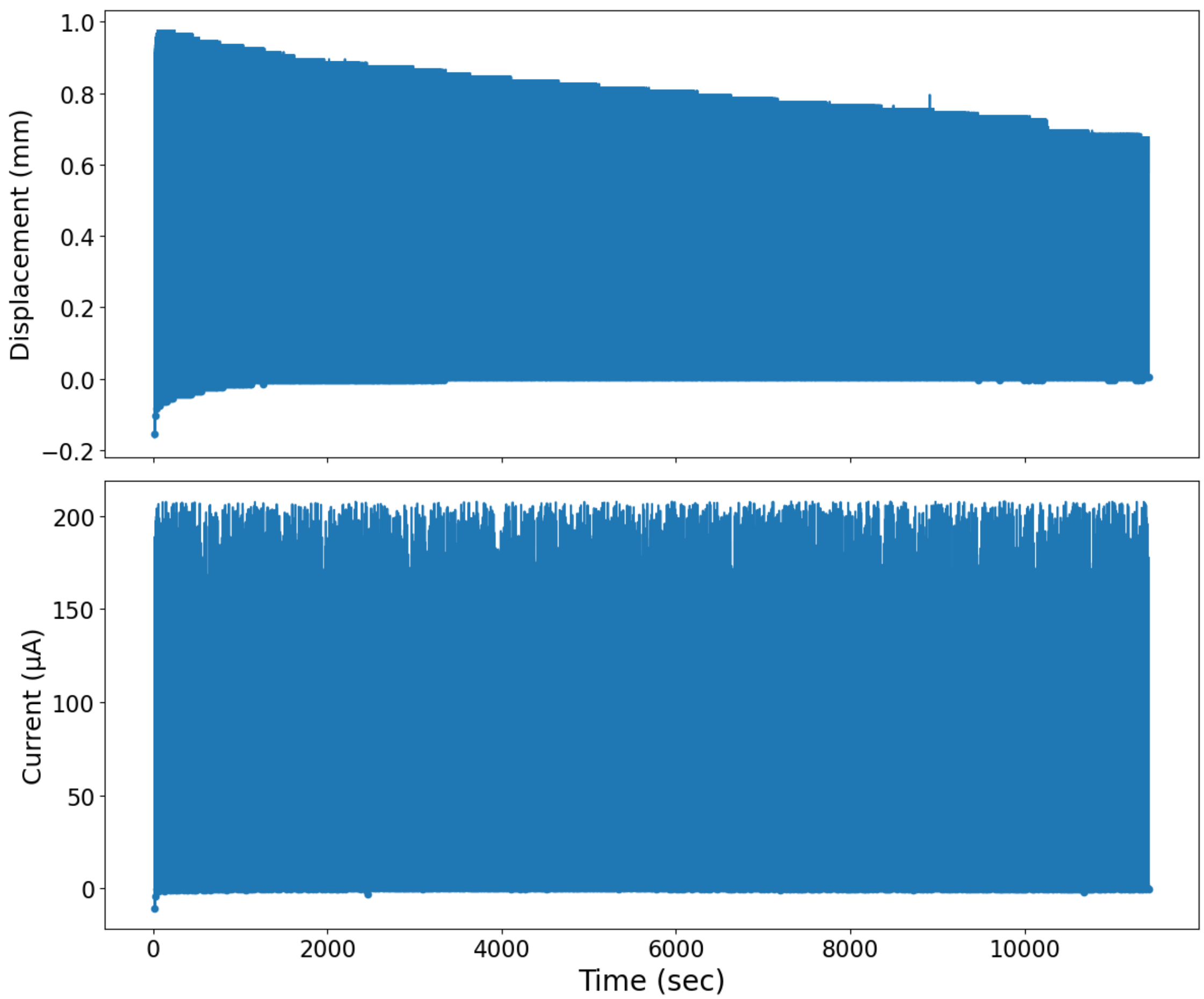

Figure S8: A DEA device actuated at 40 V/μm under a 1 Hz square-wave input (50% duty cycle) for 3 hours. Time-domain displacement and current measurements show that displacement progressively degrades during operation while the current remains relatively stable.

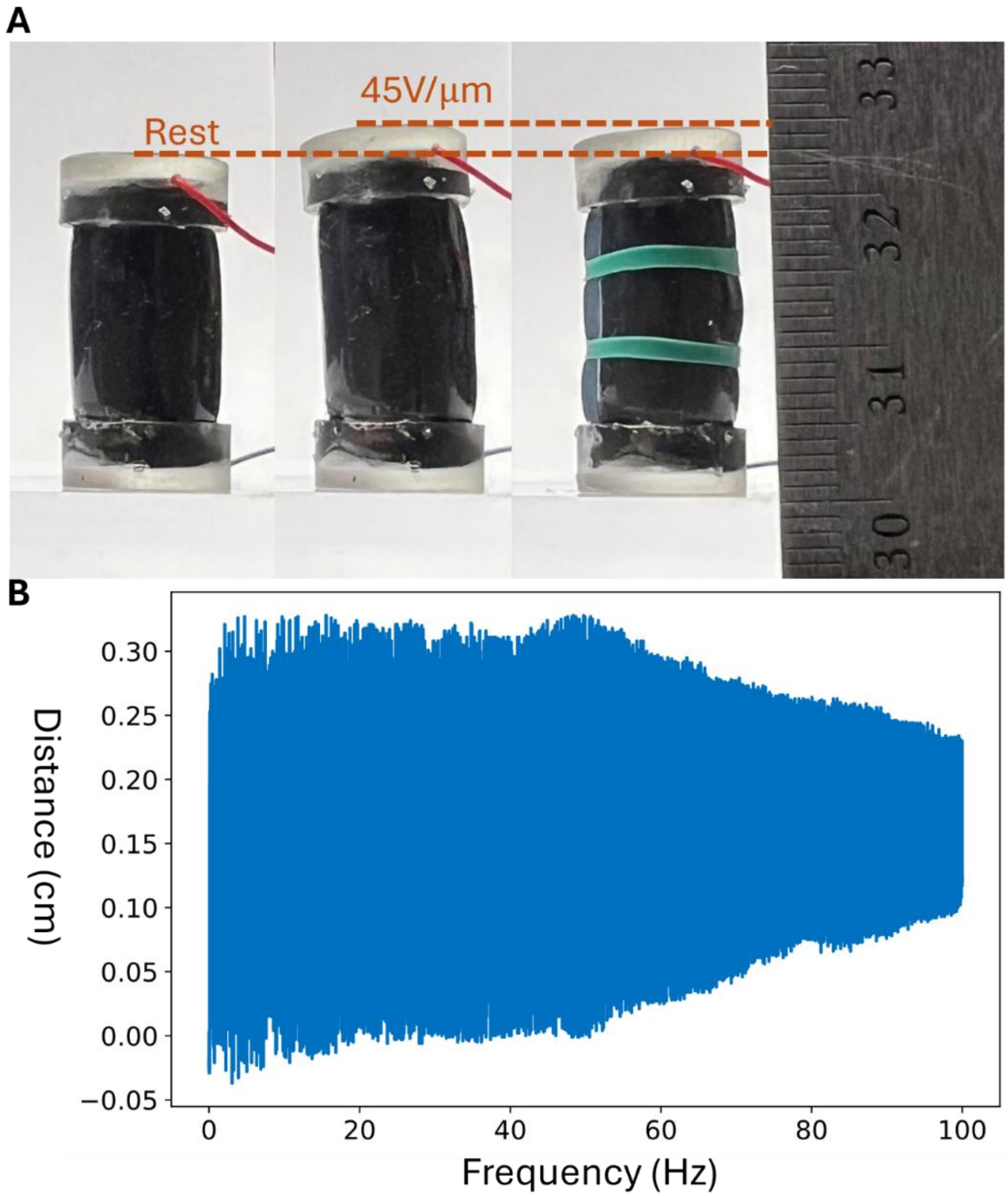

Figure S9: **Displacement performance of the scaled-up actuator.** (A) Comparison of DEA actuators with and without ribbon reinforcement at 42 $V/\mu m$. No significant difference is observed between the two configurations, both achieving about 2 mm displacement (8.7% axial strain). (B) Displacement versus frequency response of the scaled-up actuator at 45 $V/\mu m$, obtained using a sweep signal ranging from 1 Hz to 100 Hz over 100 seconds.

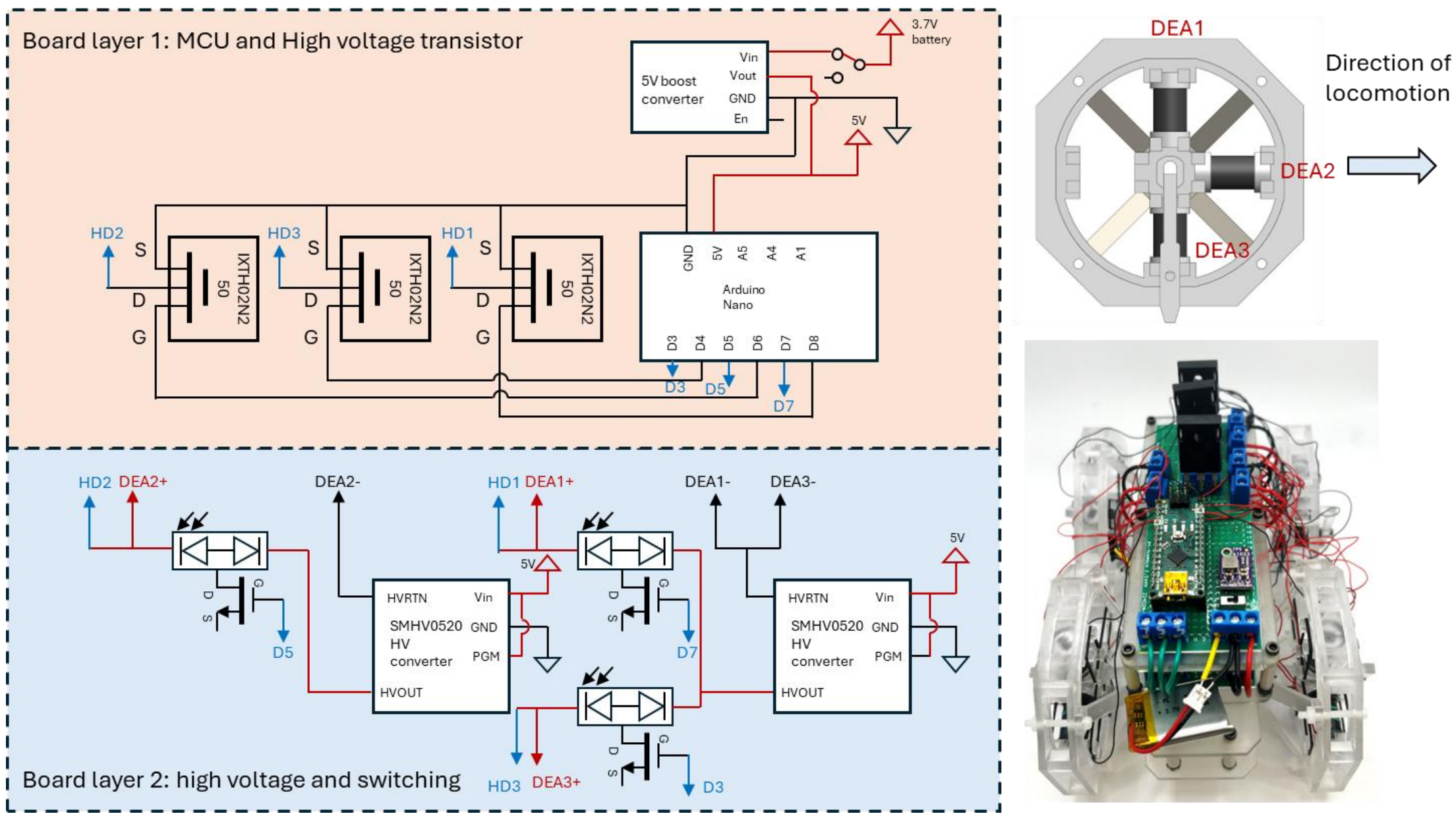

Figure S10: **Circuit diagram for untethered quadruped robot power electronics.** The top layer of the board consists of MCU, battery voltage converter, and high voltage MOSFETs for discharge. The bottom layer of the board consists of two high voltage converters (HVM SMHV0520) and 3 high voltage opto-couplers (HVM OR-100).

Supporting video files are available upon request.

Caption for Movie S1. Motor stage of the testing robot in action This video shows top and side views of the testing robot switching from displacement measurement to force measurement and then back to displacement. The rotational stepper motor moves the actuator between the two measurement positions, and the linear stepper carrying the force sensor clamps onto the actuator using force feedback. When switching back, both the linear and rotational motors return to their original positions.

Caption for Movie S2. Animation of the walking cycle of a base locomotion robot The analytical modeling of this system is in the Supplementary Text.

Caption for Movie S3. Operation of different robot configurations. The robot configurations include (i) base locomotion unit, (ii) double unit, (iii) quadruped walking robot.

Caption for Movie S4. Robot operation under different frequencies and payload. The walking speeds of a base locomotion unit, a double unit, and a quadruped robot are compared at 1 Hz and at their respective resonant frequencies. A quadruped robot carrying payload operates at its resonant frequency of 6 Hz.

References and Notes

1. D. Kühnel, F. B. Albuquerque, V. Py, H. Shea, Automated test setup to quantify the lifetime of dielectric elastomer actuators under a wide range of operating conditions. *Smart Materials and Structures* **30** (6), 065020 (2021).

2. M. Matysek, P. Lotz, H. F. Schlaak, Lifetime investigation of dielectric elastomer stack actuators. *IEEE Transactions on Dielectrics and Electrical Insulation* **18** (1), 89–96 (2011).

3. S. Jiang, C. Tang, X.-J. Liu, H. Zhao, Long-Life-Cycle and Damage-Recovery Artificial Muscles via Controllable and Observable Self-Clearing Process. *Advanced Engineering Materials* **24** (4), 2101017 (2022).

4. D. Bruch, Multifunctional test rig for accelerated assessment of electromechanical characteristics and failure mechanisms of dielectric elastomer transducers (2024).

5. H. Zhao, *et al.*, Compact dielectric elastomer linear actuators. *Advanced functional materials* **28** (42), 1804328 (2018).

6. M. Duduta, R. J. Wood, D. R. Clarke, Multilayer dielectric elastomers for fast, programmable actuation without prestretch. *Advanced Materials* **28** (36), 8058–8063 (2016).
